# Reprogrammed lipid metabolism protects inner nuclear membrane against unsaturated fat

**DOI:** 10.1016/j.devcel.2021.07.018

**Published:** 2021-09-27

**Authors:** Anete Romanauska, Alwin Köhler

**Affiliations:** 1 Max Perutz Labs, University of Vienna and Medical University of Vienna, Vienna Biocenter Campus (VBC), Dr. Bohr-Gasse 9/3, 1030 Vienna, Austria

**Keywords:** nuclear envelope, lipid metabolism, unsaturated fatty acids, inner nuclear membrane, lipid droplets, seipin, phosphatidic acid, lipid biosensors, endoplasmic reticulum, Mga2/Ole1

## Abstract

The cell nucleus is surrounded by a double membrane. The lipid packing and viscosity of membranes is critical for their function and is tightly controlled by lipid saturation. Circuits regulating the lipid saturation of the outer nuclear membrane (ONM) and contiguous endoplasmic reticulum (ER) are known. However, how lipid saturation is controlled in the inner nuclear membrane (INM) has remained enigmatic. Using INM biosensors and targeted genetic manipulations, we show that increased lipid unsaturation causes a reprogramming of lipid storage metabolism across the nuclear envelope (NE). Cells induce lipid droplet (LD) formation specifically from the distant ONM/ER, whereas LD formation at the INM is suppressed. In doing so, unsaturated fatty acids are shifted away from the INM. We identify the transcription circuits that topologically reprogram LD synthesis and identify seipin and phosphatidic acid as critical effectors. Our study suggests a detoxification mechanism protecting the INM from excess lipid unsaturation.

## Highlights

• Biosensors detect lipid saturation dynamics of INM
• Increased lipid unsaturation induces LDs at ONM, but not at INM
• Opposing transcription circuits reprogram LD synthesis across the NE
• LDs detoxify unsaturated lipids to maintain INM integrity

## Graphical abstract

**Figure:**
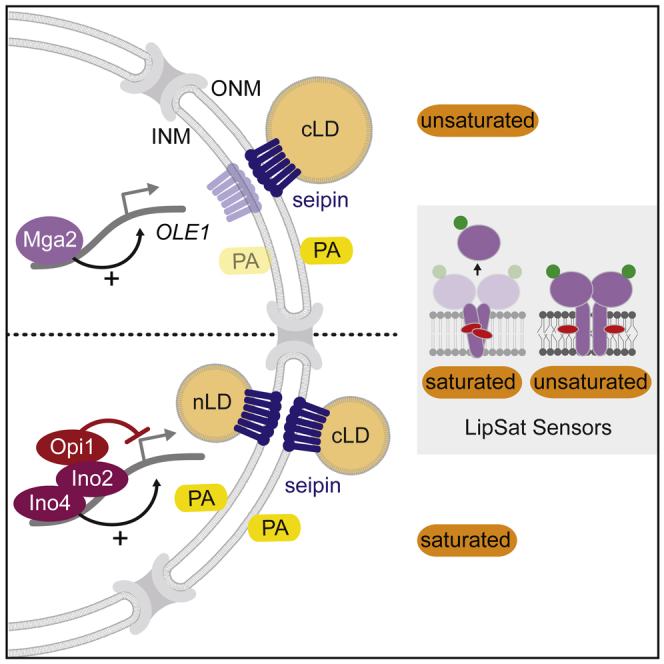

## Teaser

Romanauska and Köhler demonstrate that increased lipid unsaturation triggers a reprogramming of lipid storage metabolism across the nuclear envelope to protect the inner nuclear membrane from excess unsaturated fat. Cells induce lipid droplet formation from the outer nuclear membrane and ER, whereas lipid droplet formation at the inner nuclear membrane is suppressed.

## Introduction

The membranes of different organelles vary considerably in lipid composition and hence functionality (Bigay and Antonny, 2012; van Meer et al., 2008). The endoplasmic reticulum (ER) is a complex organelle of highly specialized subdomains comprising the nuclear envelope (NE) and the peripheral ER. The peripheral ER consists of cytoplasmic cisternae, tubules, and a plasma-membrane-associated domain in yeast (West et al., 2011). The outer nuclear membrane (ONM) and peripheral ER produce glycerophospholipids (or phospholipids in short; PL) for membrane growth, and triacylglycerol (TAG) to stockpile energy (Carman and Han, 2011). The inner nuclear membrane (INM) is in direct contact with the genome, and its point of contact with the ONM and ER is at nuclear pore complexes (Ungricht and Kutay, 2017). Active lipid metabolism at the INM enables cells to store fatty acids (FAs) in nuclear lipid droplets (nLDs) (Barbosa et al., 2019; Romanauska and Köhler, 2018). As a result of its lipid metabolism, the INM has a distinct lipid composition compared with the ONM featuring high levels of diacylglycerol, a precursor for both PL and TAG synthesis. How cells sense and adjust the lipid properties of the INM in various metabolic states is a key open question.

Fatty acyl chains, esterified in glycerophospholipids, form the hydrophobic barrier of biological membranes and determine their viscosity, thickness, water permeability, and bending rigidity (Ernst et al., 2016). Lipids with saturated acyl chains are packed more tightly and tend to form non-fluid gel phases; mono- and polyunsaturated acyl chains have kinked shapes, which fluidize bilayers. The collective biophysical properties of membranes also profoundly affect membrane-embedded proteins in their structure, activity, and signaling behavior. The fatty acyl chain profile of membranes reflects a balance between endogenous FA synthesis, recycling of FAs from lipid breakdown, and FA uptake from the exterior. Nothing is known about the fatty acyl chain composition of the INM and, hence, how INM lipid packing and viscosity, or its reciprocal, fluidity, are regulated.

**Figure 1:**
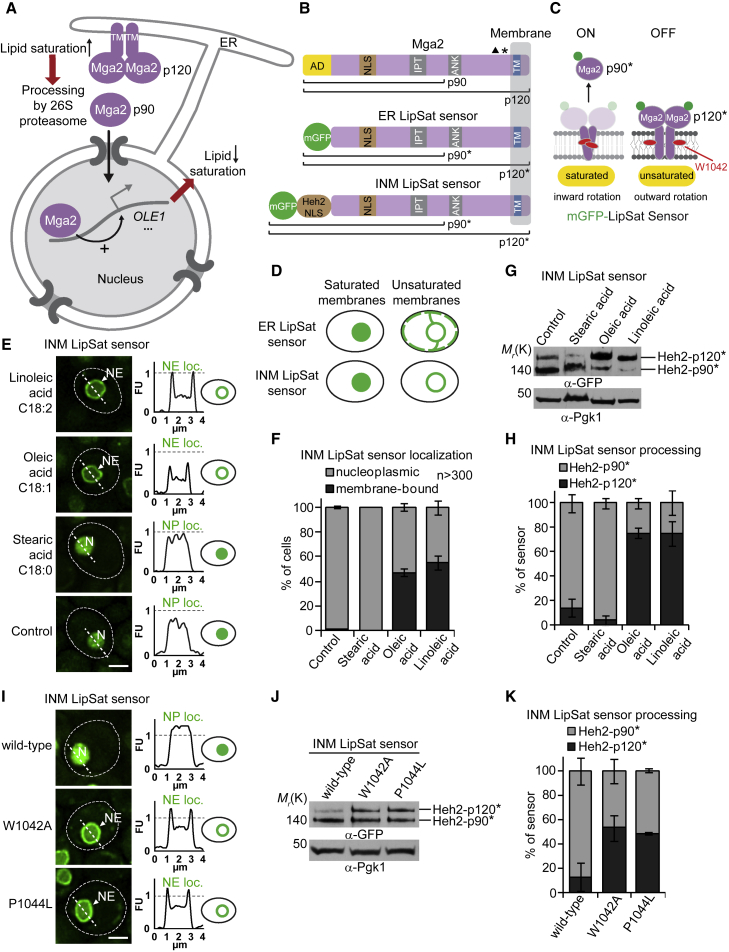
The INM dynamically responds to exogenous FAs with various degree of saturation (A) Model of the *OLE1* pathway. Homodimers of Mga2 and Spt23 (not shown) are embedded in the ER as inactive precursors (p120). Transmembrane helices (TM) sense lipid saturation/unsaturation by a conformational change. When lipid saturation decreases, Mga2 becomes ubiquitinated by the E3 Rsp5, partially processed by the proteasome and is mobilized by Cdc48 (not shown). The soluble transcription factor (p90) is imported into the nucleus, where *OLE1* transcription is activated. (B) Domain organization of wild-type Mga2 and the lipid saturation (LipSat) sensors. p120 (120 kDa) designates unprocessed Mga2, p90 (90 kDa) the processed form. LipSat sensors lack the transcriptional AD and carry an N-terminal mGFP. The full-length and processed versions of the LipSat sensors are termed p120^∗^ and p90^∗^, respectively. The NLS of the INM-resident transmembrane protein Heh2 was appended to the INM LipSat sensor for nuclear import by lateral membrane diffusion. In contrast, the endogenous NLS of Mga2 promotes import of the soluble, processed p90 fragment. IPT, immunoglobulin-like/plexins/transcription factors domain required for dimerization; ANK, ankyrin repeats; TM, transmembrane domain. Triangle indicates Rsp5-binding site, asterisk depicts multiple ubiquitination sites. (C) LipSat sensing is based on the Mga2 mechanism. A conserved tryptophane (W1042) transduces the membrane’s saturation state into an inward or outward rotational movement of the transmembrane helices. When saturated lipids increase, the sensor is activated and released from the membrane (ON). In contrast, unsaturated membranes do not trigger processing, resulting in membrane-bound LipSat sensors (OFF). Note that the E3 ligase Rsp5, the unfoldase Cdc48 and the 26S proteasome are present in both cytoplasm and nucleus allowing sensor processing in both compartments. (D) Cartoon of predicted LipSat sensor localizations. Dashed green line beneath plasma membrane depicts peripheral ER. (E) Live imaging of *mga2*Δ cells expressing the plasmid-based INM LipSat sensor supplemented with the indicated fatty acids (16 mM). Sensor fluorescence intensity was quantified across a line spanning the nucleus. For comparison the FU value 1 is marked with a horizontal dashed line. Cell contours are marked by a dashed white line. Arbitrary fluorescence units, FU; nucleus, N; nuclear envelope, NE; nucleoplasmic localization, NP loc; nuclear envelope localization, NE loc. Scale bar, 2 μm. (F) Quantification of INM LipSat sensor localization in (E). Phenotypes were classified as membrane bound or nucleoplasmic LipSat sensor. Mean value and standard deviation are depicted. n = number of analyzed cells for each condition from 3 biological replicates. (G) Immunoblotting analysis of INM LipSat sensor processing. Samples were taken from cell cultures used in (E). Heh2-p120^∗^ is membrane bound, Heh2-p90^∗^ is processed and soluble. Note that the GFP-tagged Heh2-p120^∗^/p90^∗^ fragments have a higher molecular weight than p120/p90. Pgk1 (3-phosphoglycerate kinase) serves as a loading control. (H) Quantification of INM LipSat sensor processing in (G). The percentage of Heh2-p120^∗^ and Heh2-p90^∗^ relative to total amount of sensor was quantified. The mean value and standard deviation from 3 biological replicates are depicted. (I) Live imaging of *mga2Δ* cells expressing the wild-type or mutant INM LipSat sensors. The conserved P1044 (aa position refers to full-length Mga2) is thought to provide conformational flexibility to the transmembrane helices during their relative rotations and facilitates the intimate interaction of two conserved W1042 residues in the dimer interface (see Figure 1C). Sensor fluorescence intensity was quantified across a line spanning the nucleus. For comparison the FU value 1 is marked with a horizontal dashed line. Arbitrary fluorescence units, FU; nucleus, N; nuclear envelope, NE; nucleoplasmic localization, NP loc; nuclear envelope localization, NE loc. Scale bar, 2 μm. (J) Immunoblotting analysis of INM LipSat sensor processing in (I). Pgk1 serves as a loading control. (K) Quantification of INM LipSat sensor processing in (J). The percentage of Heh2-p120^∗^ and Heh2-p90^∗^ relative to total amount of sensor was quantified. The mean value and standard deviation from 3 biological replicates are depicted.

In budding yeast, Ole1 is the sole enzyme that can introduce a double bond into fatty acyl chains (Martin et al., 2007). There is no opposing enzymatic activity making this reaction irreversible. Ole1 is located in the ER and specifically introduces a double bond at the C9 position. The expression of Ole1 is controlled by the cellular lipid acyl chain composition and is strongly reduced when unsaturated fatty acids (UFAs) are abundant (Figure 1A). This feedback control depends on the sensor proteins Mga2 and Spt23, which are produced as homo-dimeric, ER-bound, inactive precursors of transcription factors (Covino et al., 2016; Hoppe et al., 2000). The crucial element for UFA sensing is Mga2’s transmembrane helix (Figure 1A), which harbors specific sensory residues embedded deep in the lipid bilayer (Covino et al., 2016). The transmembrane helices continuously explore alternative rotational states. Loose lipid packing (high UFA content) stabilizes conformations where two sensory tryptophan residues point away from the dimer interface toward the lipid environment. Tight lipid packing (low UFA content) stabilizes alternative rotational conformations with the sensory tryptophans facing each other in the dimer interface. Thus, Mga2 operates via a rotation-based mechanism: the transmembrane helices sense the membrane state and through conformational changes transduce this signal to Mga2’s ubiquitination sites (Covino et al., 2016). When unsaturated lipids are scarce and the lipid packing density is high, Mga2 and Spt23 are ubiquitinated by the E3 ligase Rsp5, partially processed by the proteasome to release the active transcription factor, and imported into the nucleus via a cryptic nuclear localization sequence (NLS) to increase Ole1 expression (Piwko and Jentsch, 2006; Rape et al., 2001) (Figures 1A and 1B). In contrast, when unsaturated lipids are abundant and the lipid packing density is low, these transcription factors remain tethered to the ER.

Nutrient-derived FAs can be toxic, leading to ER stress, proliferation of ER membranes, and finally lipoapoptosis (Garbarino et al., 2009; Petschnigg et al., 2009). UFAs seem to be particularly toxic and are mainly channeled into neutral lipids with subsequent biogenesis of LDs. LDs appear to be critical as a storage space for potentially harmful UFAs (Fakas et al., 2011; Velázquez et al., 2016), but the molecular mechanisms of UFA detoxification are poorly understood. A particular problem is to separate the pleiotropic effects of nutrient overload from the specific effects of acyl chain saturation. Here, we have employed a targeted genetic strategy to introduce a single double bond in FAs without nutrient overload.

UFAs probably reach the INM by lateral diffusion from the ONM. If and how the INM controls its saturation status is unknown and of potentially significant physiologic consequences considering its specialized proteome and intimate contact with the genome. We previously observed that oleic acid supplementation (a monounsaturated FA) or mutations in Cds1, a key enzyme in PL synthesis, induced LD formation from both the INM and ONM, creating nuclear and cytoplasmic LDs (nLDs and cLDs), respectively (Romanauska and Köhler, 2018). We therefore sought to explore whether LD formation at the NE contributes to detoxifying unsaturated acyl chains from the INM.

In this study, we have developed tools to directly examine lipid (un-)saturation at the INM in living cells. We provide evidence that cells orchestrate lipid metabolism across the NE by channeling potentially harmful UFAs into cytoplasmic, but not nuclear, LDs, thus protecting the INM and the nucleus from UFA-mediated lipotoxicity.

## Results

### Biosensors report nutrient-dependent lipid saturation dynamics of INM

Since the INM is capable of lipid metabolism, we asked how this specialized membrane territory, adjacent to the genome, responds to changes in lipid saturation. We developed genetically encoded, fluorescently labeled lipid saturation (LipSat) sensors, which measure fatty acyl chain saturation in phospholipids specifically at the INM, or, across the entire ER/NE network. These LipSat sensors are based on *S.cerevisiae* Mga2 (Figure 1A) and are targeted to the INM or the ER/NE by appending or omitting, respectively, the NLS of the INM transmembrane protein Heh2 (Meinema et al., 2011) (Figure 1B). The Heh2 NLS directs lateral diffusion from the ONM to the INM via the nuclear pore complex. This import mechanism is distinct from that of the processed, soluble part of Mga2, which is mediated by a cryptic NLS (Figure 1B). We deleted the transcriptional activation domain (AD) of Mga2 to uncouple UFA sensing from Ole1 transcription (Hoppe et al., 2000). We expected an mGFP-labeled fragment of the LipSat sensors (p90^∗^) to be clipped off the membrane by the proteasome when membrane lipids are saturated (i.e., UFA levels are low) (Figures 1B–1D) and to accumulate in the nucleoplasm due to its cryptic NLS. In contrast, the LipSat sensors would remain membrane bound (p120^∗^) when membrane lipids are unsaturated (i.e., cellular UFA content is high) (Figures 1C and 1D). The sensors were expressed in *mga2*Δ cells to avoid hetero-dimerization with wild-type Mga2. They were expressed at near endogenous levels and did not affect cell growth under the conditions tested (Figures S1A and S1B).

The control *mga2Δ* cells, which have reduced Ole1 activity (Martin et al., 2007), exhibited a largely processed LipSat sensor, consistent with a lack of unsaturated fatty acyl chains (Figures 1E–1H). To confirm that the LipSat sensors faithfully detect the degree of fatty acyl chain saturation in membranes, we supplemented the media with saturated stearic acid (C18:0), monounsaturated oleic acid (C18:1) or di-unsaturated linoleic acid (C18:2). These FAs are metabolized and incorporated into PLs, hence altering their degree of saturation. As a quantitative readout, we determined the amount of nucleoplasmic mGFP-p90^∗^ versus the membrane-bound mGFP-p120^∗^ protein by fluorescence microscopy and by immunoblotting. The INM LipSat sensor was found mostly unprocessed at the INM when cells were supplemented with linoleic acid (C18:2) or oleic acid (C18:1) (Figures 1E–1H), as expected for increased UFA levels. By comparison, cells supplemented with fully saturated stearic acid (C18:0) showed enhanced sensor processing and accumulation of mGFP-p90^∗^ in the nucleoplasm (Figures 1E–1H). Hence, nutrient-derived unsaturated acyl chains can accumulate in the INM. Wild-type cells contained more unprocessed sensor at the INM than *mga2Δ* cells, indicating that Ole1 unsaturase activity increases UFAs at the INM, but not to the same extent as the exogenously supplied UFAs (Figures S1C–S1F).

Importantly, mutating the sensory W1042 and P1044 residues in the transmembrane helix of the INM LipSat recapitulates the sensing defect that was previously reported for endogenous Mga2 (Covino et al., 2016). This evidence supports the notion that the Mga2-derived biosensors use the same rotation-based UFA sensing mechanism as endogenous Mga2 (Figures 1I–1K).

In parallel, we characterized the global ER LipSat sensor, which localized to both the peripheral ER and the NE. This sensor’s response to UFAs mirrored the behavior of the INM sensor: it remained unprocessed and membrane bound when UFA levels were increased (i.e., linoleic acid) but became processed and nucleoplasmic when UFA levels decreased (i.e., stearic acid) (Figures S1G–S1J). The INM LipSat sensor responded more strongly to changes in fatty acyl chain saturation levels compared with the global ER LipSat sensor (see Figures 1H and S1J). This might reflect a physiologically relevant hypersensitivity of the INM toward lipid unsaturation or a greater accumulation of unsaturated lipids in this membrane territory. In sum, our lipid saturation sensors can detect nutrient-induced fatty acyl chain profiles of the INM or the entire ER network.

### Biosensors detect consequences of Ole1 activity at INM

**Figure 2:**
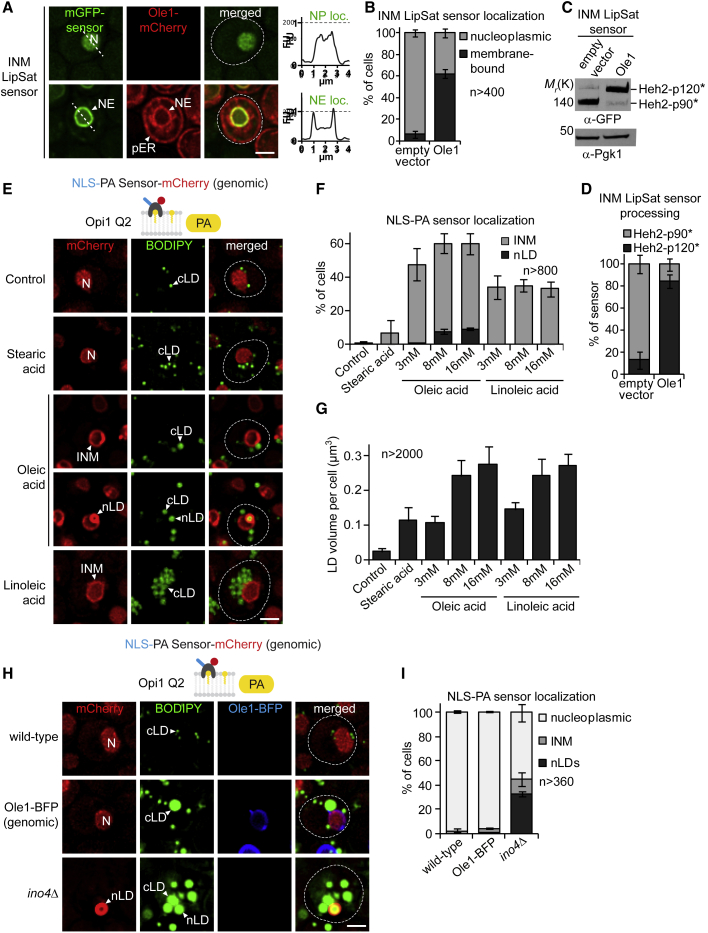
Ole1 overexpression increases UFA but not PA levels at the INM (A) Live imaging of cells expressing the INM LipSat sensor together with Ole1-mCherry (bottom panel) or an empty vector (top panel). Ole1-mCherry was expressed from the strong *GPD (TDH3)* promoter. Plasmids were transformed into *mga2Δ* cells. Sensor fluorescence intensity was quantified across a line spanning the nucleus. For comparison the FU value 1 is marked with a horizontal dashed line. Arbitrary fluorescence units, FU; nucleus, N; peripheral endoplasmic reticulum, pER; nuclear envelope, NE; nucleoplasmic localization, NP loc; nuclear envelope localization, NE loc. Scale bar, 2 μm. (B) Quantification of INM LipSat sensor localization in (A). Phenotypes were classified as membrane bound or nucleoplasmic. Mean value and standard deviation depicted. n = number of analyzed cells for each condition from 3 biological replicates. (C) Immunoblotting analysis of INM LipSat sensor processing in (A). Note that the GFP-tagged Heh2-p120^∗^/p90^∗^ fragments have a higher molecular weight than p120/p90. Pgk1 serves as a loading control. (D) Quantification of INM LipSat sensor processing in (C). The percentage of Heh2-p120^∗^ and Heh2-p90^∗^ relative to total amount of sensor was quantified. The mean value and standard deviation from 3 biological replicates are depicted. (E) Live imaging of genomically integrated NLS-PA-mCherry sensor expressed in wild-type cells, which were supplemented with the indicated fatty acids (each 16 mM dissolved in 1.5% Brij L23 solution). The NLS-PA-mCherry sensor contains the Q2 domain of the *S. cerevisiae* transcription factor Opi1 that specifically recognizes phosphatidic acid (PA). LDs are stained with the BODIPY dye. Nucleus, N; inner nuclear membrane, INM; nuclear lipid droplet, nLD; cytoplasmic lipid droplet, cLD. Scale bar, 2 μm. (F) Quantification of NLS-PA-mCherry localization as observed in (E). Additional fatty acid concentrations were also quantified. n = number of analyzed cells from 3 biological replicates are depicted. Using t test, a statistically significant difference for the percentage of nLDs was verified between 16 mM oleic and 16 mM linoleic acid; and between 3 and 8 mM oleic acid. 8 and 16 mM oleic acid were not significantly different. (G) Quantification of total LD volume per cell in (E). Additional fatty acid concentrations were also quantified. LD volumes were measured as described in STAR Methods. n = number of analyzed cells from at least 3 biological replicates. Using t test, no statistically significant difference of LD volume per cell between 16 mM oleic and 16 mM linoleic acid, or between 8 mM oleic acid and 8 mM linoleic acid was found. (H) Live imaging of the indicated strains expressing genomically integrated NLS-PA-mCherry sensor. LDs are stained with the BODIPY dye. Genomically integrated BFP-tagged Ole1 was overexpressed from the *GPD* promoter. Nucleus, N; nuclear lipid droplet, nLD; cytoplasmic lipid droplet, cLD. Scale bar, 2 μm. (I) Quantification of NLS-PA-mCherry sensor localization as observed in (H). n = number of analyzed cells from 3 biological replicates.

Budding yeast cells take up various exogenous UFAs, including UFAs they cannot produce themselves like linoleic acid (C18:2) (Martin et al., 2007). The broad substrate specificity of acyl-CoA synthetases may explain the ability of yeast to incorporate non-native, polyunsaturated FAs from their environment (Black and DiRusso, 2007). In contrast, Ole1 (human SCD1), the sole fatty acid desaturase in yeast, only introduces a single double bond at the C9 position of palmitic (C16:0) and stearic (C18:0) acid. To test whether the LipSat sensors detect this endogenous unsaturase activity, we expressed Ole1 from a strong *GPD* promoter in *mga2Δ* cells (Figure 2A). This resulted in a largely unprocessed, INM-bound LipSat sensor demonstrating that Ole1 increases PL unsaturation of the INM (see Figures 2A and 2B for sensor localization, and Figures 2C and 2D for quantitative immunoblotting). In contrast, the LipSat sensor was processed in *mga2Δ* control cells containing an empty vector, indicating high INM saturation levels (Figures 2A–2D). A similar sensor response was seen for the global ER LipSat sensor (Figures S2A–S2D). Thus, Ole1 activity directly affects the INM and ER/ONM acyl chain profile.

To verify that the INM LipSat sensor measures lipid saturation of the INM rather than the ER/ONM, we confirmed that sensing and proteasomal processing (Figure 1A) indeed take place at the INM. We generated LipSat sensors tagged with mGFP and mCherry at the N and C terminus, respectively (Figure S1K), and overexpressed Ole1 to increase cellular UFA content. If the sensor was processed at the ER, the mCherry-containing transmembrane helix should remain in the ER, whereas the mGFP-p90^∗^ fragment is targeted to the nucleoplasm (Figures S1L and S1M). Importantly, the mCherry-transmembrane helix of the INM LipSat sensor was not detected at the peripheral ER, consistent with the idea that UFA sensing and sensor processing take place directly at the INM (Figure S1N). In contrast, the mCherry-transmembrane helix of the ER LipSat sensor was detected at the ER (Figure S1N). Finally, we ascertained that the LipSat sensors are inert, such that sensing at the ONM/ER does not influence UFA accumulation at the INM and vice versa. The INM LipSat sensor, co-expressed with an ER LipSat sensor, showed a similar UFA response when compared with cells that expressed the INM LipSat or ER LipSat sensor alone. Hence, the two sensors, expressed in different membrane compartments do not interfere with each other (Figures S2E–S2G). Taken together, the development of compartment-specific LipSat biosensors enabled us to show that the INM experiences changes in PL saturation that are induced by exogenous UFAs or Ole1 activity.

### The type of nutrient FAs influences the site of LD formation

Upon FA overload, cells produce excess TAG, which is stored in LDs (Henne et al., 2020; Olzmann and Carvalho, 2019; Walther et al., 2017). In principle, LDs can be synthesized either from the ONM/peripheral ER (cLDs), or from the INM (nLDs). The decision-making behind the site of LD formation (cytoplasm versus nucleoplasm) is unknown; however, high INM phosphatidic acid (PA) levels may favor nLD production (Romanauska and Köhler, 2018). To find out how the saturation status of FAs affects INM lipid metabolism and nLD formation, we supplemented wild-type cells with equimolar amounts of SFAs (stearic acid, C18:0) and UFAs (oleic acid, C18:1 or linoleic acid, C18:2) (Figure 2E). To detect nLDs, we employed a previously established PA biosensor (NLS-Opi1 Q2-mCherry) in conjunction with the BODIPY 493/503 dye that stains neutral lipids. During nLD synthesis, this NLS-PA sensor becomes first enriched at the INM and subsequently on nLDs, which are connected to the INM by membrane bridges (Romanauska and Köhler, 2018). Without FA supplementation, and when PA levels at the INM are low (Figures 2E and 2F, control), the NLS-PA sensor exhibited a nucleoplasmic localization. Stearic acid (C18:0; 16 mM) produced a ∼4-fold increase of LD volume per cell compared with control cells without FA supplementation (Figure 2G). However, PA levels at the INM remained low (i.e., the NLS-PA sensor stayed mostly nucleoplasmic), and we did not detect nLDs. In contrast, oleic acid (C18:1; 16 mM) induced a ∼10-fold increase of LD volume per cell and generated both cLDs as well as nLDs (Figures 2E–2G). nLDs were present in ∼10% of cells under the conditions tested. Interestingly, linoleic acid, a UFA with two double bonds (C18:2; 16 mM), generated a similar amount of total LDs as oleic acid (C18:1), but essentially no nLDs, even though INM PA levels were increased. Linoleic acid failed to induce nLDs in the range of 3–16 mM, though the LD volume per cell was similar to that induced by oleic acid (Figures 2F and 2G). This result was unexpected because we assumed that the nLD/cLD ratio would remain constant irrespective of the FA class and that an increase of PA would be permissive for nLD formation. Thus, nLD synthesis does not scale with the total amount of LDs in cells but appears to be influenced by the type of FA that is supplied and additional factors besides a high INM PA content. Importantly, increasing the number of double bonds in FAs (i.e., linoleic acid) induces cLD biogenesis, yet, may suppress nLD synthesis.

### Lipid unsaturation topologically reprograms LD biogenesis at NE

An overload of cells with dietary UFAs has a dual effect as it increases both the total FA content as well as PL unsaturation (Garbarino et al., 2009). Hence, it has remained unclear to what extent lipid unsaturation per se contributes to LD production. Ole1 overexpression provided us with the opportunity to selectively increase the intracellular UFA/SFA ratio. Strikingly, this led to a massive synthesis of LDs (Figure S2H) even though cells were not overfed with nutrient FAs. The amount of newly synthesized LDs correlated positively with Ole1 protein levels and mainly affected LD volume rather than LD number per cell (Figures S2I–S2K). Hence, introducing a single double bond at the C9 position is a potent driver of LD biogenesis.

We then asked, in which region of the ER network these LDs are synthesized (i.e., INM versus ONM/peripheral ER). To do so, we determined whether LDs represent nLDs, cLDs, or both, by employing the NLS-PA sensor as an nLD marker together with BODIPY as a marker for all LDs in a cell. Notably, Ole1 overexpression increased the amount of cLDs but yielded essentially no nLDs (Figures 2H and 2I). We used *ino4Δ* cells as a benchmark for cells with a high nLD content, since blocking the Opi1-Ino2/4 transcriptional circuit is a strong stimulus for both cLD and nLD production (Romanauska and Köhler, 2018). We detected nLDs as prominent BODIPY- and PA-positive nuclear structures in ∼32% of *ino4Δ* cells (Figures 2H and 2I). In contrast, Ole1 overexpression selectively increased cLD production. Hence, our data suggest that the conversion of endogenous SFAs into UFAs by Ole1 favors cLD over nLD synthesis similar to what we had observed when exogenously providing double-unsaturated FAs.

### Mga2 directs LD biogenesis toward the cytoplasm to adjust INM saturation

**Figure 3:**
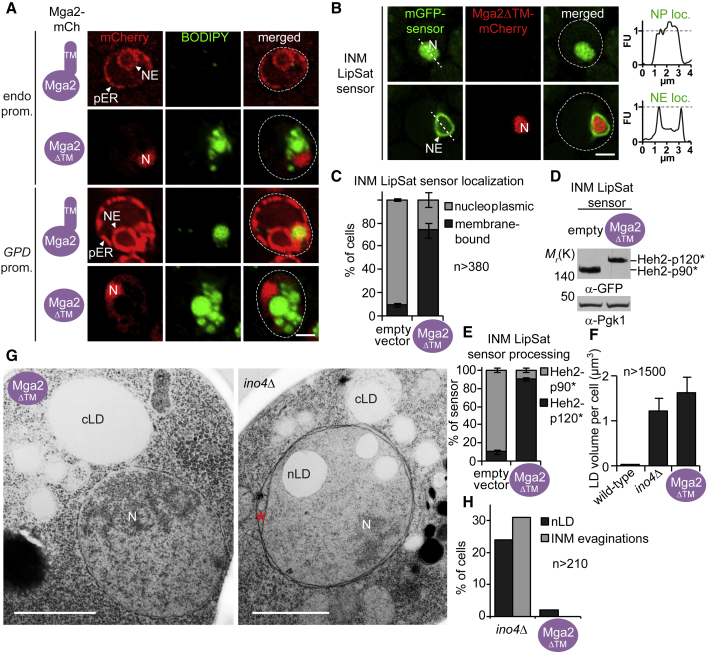
Mga2 selectively promotes cytoplasmic lipid droplet production (A) Live imaging of *mga2*Δ cells expressing plasmid-based, full-length Mga2-mCherry or Mga2-mCherry lacking the transmembrane helix (Mga2ΔTM). Mga2 variants were expressed from the endogenous *MGA2* or the strong *GPD* promoter (see also Figures S2L and S2M). LDs are stained with BODIPY. Nucleus, N; peripheral ER, pER; nuclear envelope, NE. Scale bar, 2 μm. (B) Live imaging of the INM LipSat sensor co-expressed with Mga2ΔTM-mCherry or an empty vector. Genomically integrated Mga2ΔTM-mCherry was expressed from the strong *GPD* promoter in *mga2*Δ cells. Sensor fluorescence intensity was quantified across a line spanning the nucleus. For comparison the FU value 1 is marked with a horizontal dashed line. Arbitrary fluorescence units, FU; nucleus, N; nuclear envelope, NE; nucleoplasmic localization, NP loc; nuclear envelope localization, NE loc. Scale bar, 2 μm. (C) Quantification of INM LipSat sensor localization in (B). Phenotypes were classified as membrane bound or nucleoplasmic. Mean value and standard deviation are depicted. n = number of analyzed cells for each condition from 3 biological replicates. (D) Immunoblotting analysis of INM LipSat sensor processing in (B). Note that the GFP-tagged Heh2-p120^∗^/p90^∗^ fragments have a higher molecular weight than p120/p90. Pgk1 serves as a loading control. (E) Quantification of INM LipSat sensor processing in (D). The percentage of Heh2-p120^∗^ and Heh2-p90^∗^ relative to total amount of sensor was quantified. The mean value and standard deviation from 3 biological replicates are depicted. (F) Quantification of total LD volume per cell in the indicated strains. n = number of analyzed cells from 3 biological replicates. Mean value and standard deviation are depicted. (G) Ultrastructural analysis of *ino4*Δ and Mga2ΔTM cells by TEM. Plasmid-based Mga2ΔTM was expressed from the strong *GPD* promoter in *mga2*Δ cells (see also Figure S3I). The red asterisk marks NE evaginations, which are a common feature of *ino4Δ* cells. Cytoplasmic lipid droplet, cLD; nuclear lipid droplet, nLD; nucleus, N. Scale bar, 1 μm. (H) Quantification of nLDs and NE evaginations in (G). n = number of analyzed cells.

To understand whether the balance of nLDs and cLDs is controlled by the Mga2 feedback circuit (Figure 1A), we tested whether Mga2 elicits the same phenotype as Ole1. We created an Mga2 version that lacks the ER-anchored TM domain (Mga2ΔTM) and is constitutively imported into the nucleus. Full-length Mga2, expressed from its endogenous promoter, localized to the entire ER network, and these cells contained few small LDs. In contrast, Mga2ΔTM was imported into the nucleus and induced LD production (Figure 3A; see Figure S2L for expression levels and S2M for LD quantification). This effect was also seen upon overexpression of the wild-type Mga2 from a stronger *GPD* promoter. Strikingly, the overexpression of Mga2ΔTM from the *GPD* promoter led to a massive accumulation of LDs, which turned yeast into adipocyte-like cells (Figure 3A).

Using the LipSat sensors, we confirmed that lipids of the INM and ER/ONM were highly unsaturated under these conditions (Figures 3B–3E and S3A–S3D). Overexpression of other transcriptional targets of Mga2, such as the FA elongase *ELO1*, the LD-associated fatty acyl-CoA synthetase *FAA4* (Kelley and Ideker, 2009), or the sterol-synthesis enzyme *MVD1* (see transcriptome below), did not increase cellular LDs (Figure S2N). In contrast, overexpression of Spt23, the paralog of Mga2, did induce LDs (Figure S3E). Hence, Mga2/Spt23 overexpression resembles Ole1 overexpression.

To quantitatively study the ultrastructure of LDs produced by the Mga2-Ole1 circuit, we employed transmission electron microscopy (TEM). As a control, we examined *ino4Δ* cells, which produce both nLDs and cLDs. With TEM we found nLDs in ∼23% of *ino4*Δ cells, but only in ∼2% of Mga2ΔTM-expressing cells (Figures 3G, 3H, and S3I). The TEM data are in agreement with live-cell imaging, in which we visualized the topological relationship of LDs with the NE by combining BODIPY staining with the INM marker Heh2-mCherry (Figures S3F and S3G). The *ino4*Δ cells contained nLDs in ∼18% of cells compared with only ∼5% of Mga2ΔTM-expressing cells. We previously described INM evaginations as a feature of nLD formation in *ino4Δ* cells (Romanauska and Köhler, 2018) (Figure 3H). These INM evaginations were absent in Mga2ΔTM cells (Figures 3G and 3H). Importantly, the low number of nLDs in Mga2ΔTM cells is not caused by a reduced overall LD content because Mga2ΔTM cells contain a higher LD volume per cell than *ino4*Δ cells (Figure 3F). In sum, our data indicate that cells cope with lipid unsaturation by specifically producing cLDs rather than nLDs and that this bias is mediated by the Mga2/Ole1 pathway.

### Transcriptome signatures of compartment-specific LD synthesis

**Figure 4:**
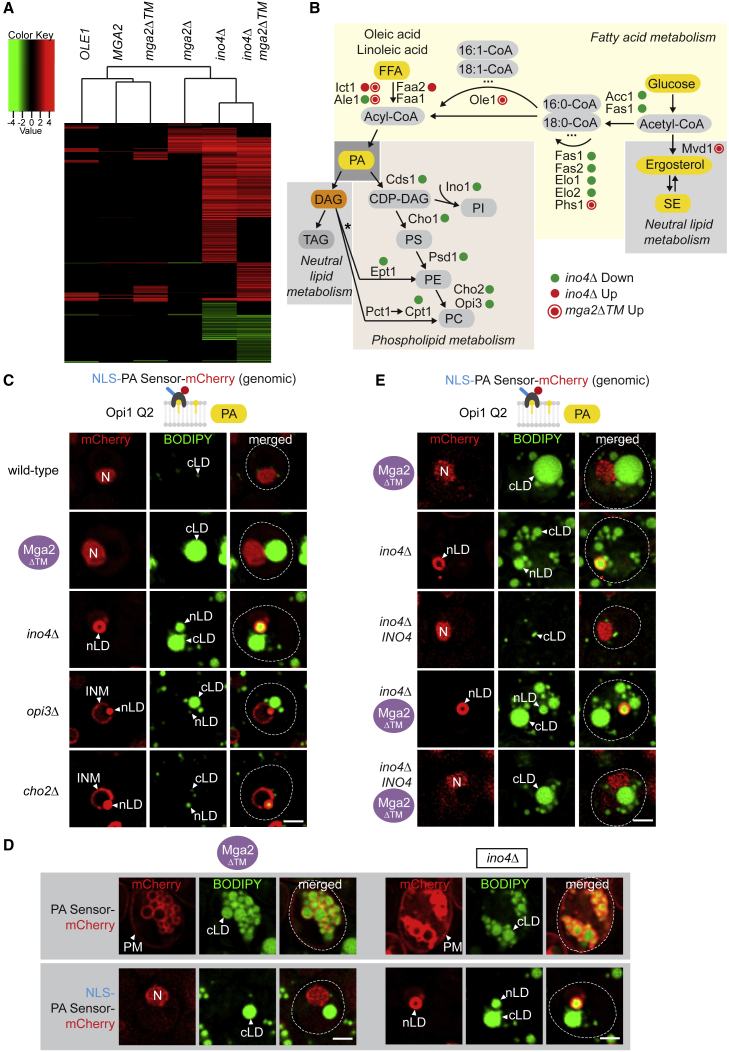
Transcriptome signatures of compartment-specific LD synthesis (A) Cluster diagram of genes with significantly altered mRNA levels (>1.5-fold) in the indicated strains. Changes in mRNA levels were compared with the wild-type strain and are depicted in red (up), green (down), or black (no change). See also Figure S4A. (B) Simplified scheme of lipid metabolism in yeast. Major pathways are color coded, and key lipid intermediates/end products are depicted. Differentially transcribed enzymes in the mutant strains are shown and marked with a green dot (down), red dot (up). See also Figure S4B for the additional mutants shown in Figure 4A. Asterisk indicates the Kennedy pathway, which uses exogenous choline and ethanolamine together with DAG to form PE and PC. (C) Live imaging of NLS-PA-mCherry sensor expressed genomically in the indicated strains (see also Figure S4E). BODIPY stains LDs. Nucleus, N; inner nuclear membrane, INM; nuclear lipid droplet, nLD; cytoplasmic lipid droplet, cLD. Scale bar, 2 μm. (D) Comparison of PA distribution in the nucleus and cytoplasm. Live imaging of the PA-mCherry sensor (cytoplasm) or NLS-PA-mCherry sensor (nucleus) expressed in *ino4*Δ and Mga2ΔTM cells. Genomically integrated Mga2ΔTM was overexpressed from the *GPD* promoter. BODIPY stains LDs. Nucleus, N; plasma membrane, PM; cytoplasmic lipid droplet, cLD; nuclear lipid droplet, nLD. Scale bar, 2 μm. (E) Live imaging of NLS-PA-mCherry sensor in the indicated strains (see also Figures S4H and S4I). BODIPY stains LDs. Nucleus, N; nuclear lipid droplet, nLD. Scale bar, 2 μm.

To understand the mechanism by which cells decide whether to synthesize LDs from the INM or not, we sought to identify transcripts whose abundances specifically differ when nLDs as well as cLDs or only cLDs are formed. We performed gene expression profiling by RNA sequencing (RNA-Seq) with strains that are inhibited in the Opi1/Ino2/4 or Mga2 pathway (i.e., *ino4Δ* versus *mga2*Δ), hyperactive in the Mga2 pathway (i.e. *OLE1, MGA2*, and *mga2ΔTM* overexpression), or have mutations in both pathways (i.e., *ino4Δ mga2ΔTM*). This allows to distinguish conditions with prominent nLDs and cLDs (e.g., *ino4Δ*) from conditions with cLDs but no nLDs (e.g., *mga2ΔTM* overexpression). These datasets were compared with each other by hierarchical clustering of transcriptome changes using a stringent 1.5-fold cutoff and a false discovery rate of p < 0.05 (Figure 4A). Conditions without nLDs (*OLE1*, *MGA2*, or *mga2ΔTM* overexpression) clustered together, and the *ino4Δ mga2ΔTM* double mutant clustered with *ino4Δ*, which produces nLDs. Deletion of *INO4* upregulated 678 genes and downregulated 183 genes (Figures 4A and S4A). ∼7% of these genes were involved in lipid metabolism. *mga2Δ* cells exhibited 142 differentially expressed genes including the ubiquitin gene *UBI4*, consistent with the upregulation of the unfolded protein response (UPR) upon increased lipid saturation and lipid bilayer stress (Surma et al., 2013). In contrast, Mga2ΔTM overexpression produced fewer differentially regulated transcripts (120 upregulated, 6 downregulated; ∼9% lipid metabolism genes), which were similar to Ole1- and Mga2-overexpressing cells. We did not observe a broad overlap between genes that are affected by the Opi1/Ino2/4 or Mga2/Ole1 pathway consistent with their distinct roles in lipid metabolism (Figure 4A).

We mapped the up- and downregulated genes onto a simplified lipid metabolism diagram, which contains major metabolic branch points (Figures 4B and S4B). A striking difference between the transcriptomes of Mga2ΔTM-overexpressing cells (cLDs) and *ino4*Δ cells (cLDs and nLDs) is the fact that enzymes involved in PL synthesis, for example, the phosphatidylethanolamine methyltransferase *CHO2* or the phospholipid methyltransferase *OPI3* are downregulated specifically in *ino4*Δ cells (Figures 4B and S4C). In contrast, Mga2ΔTM overexpressing cells showed an upregulation of the Ole1 desaturase and Mvd1, an enzyme involved in sterol biosynthesis, whereas the expression of PL synthesis factors remained largely unchanged (Figures 4B and S3H). Inhibition of the Opi1/Ino2/4 pathway via *INO4* deletion had a dominant effect in Mga2ΔTM cells because genes involved in PL synthesis were again suppressed in the double mutant (Figures 4A and S4B). Collectively, these findings raise the possibility that nLD production in *ino4*Δ cells is accomplished, in part, by downregulating enzymes involved in PL synthesis. We hypothesized that this leads to an accumulation of PA, which is channeled into lipid storage metabolism. Indeed, cellular PA levels were increased (Figure S4D), which should lead to the depletion of phosphatidylcholine (PC), a major end product of phospholipid synthesis (Figure 4B). We then examined PA levels directly at the INM in *cho2Δ* or *opi3Δ* cells. Using the NLS-bearing PA sensor (Figures 4C and S4E), we found that both mutants had increased INM PA levels and ∼10% nLDs. Although the penetrance of the nLD phenotype is not as strong as in *ino4*Δ cells, this shows that even single mutations in the PL branch can partially phenocopy nLD formation in *ino4Δ* cells (Figure 4A). As expected, Mga2ΔTM-overexpressing cells exhibited a nucleoplasmic NLS-PA sensor localization, indicating low INM PA levels (Figures 4C, S4D, and S4E). Therefore, Mga2ΔTM cells may shift LD production to the cytoplasm because the active PL branch consumes PA that is not available for nLD production at the INM.

To directly detect PC levels at the INM, we examined the localization of the cholinephosphate cytidylyltransferase Pct1 (mammalian CCT), which synthesizes PC via the condensation of choline and DAG in the Kennedy pathway (Figure 4B, asterisk). A fraction of Pct1 is intranuclear and membrane binding occurs when PC levels are low (Haider et al., 2018). Using Pct1-mCherry binding to the INM as a proxy of PC content, we found that INM PC levels are low in *cho2Δ* and *opi3Δ* cells, which have a deficiency in converting PA into PC (Figures S4F and S4G). In contrast, Mga2ΔTM-expressing cells had wild-type-like PC levels at the INM, indicating that PA to PC conversion is largely intact.

In sum, gene expression profiling allowed us to pinpoint critical factors for a topological reprogramming of LD synthesis across the NE. Both transcriptional circuits converge on increasing global LD production (Figure 3F). However, they differ substantially with respect to their effects on the cellular compartment, in which LDs are made. The global lipid synthesis pathway Opi1/Ino2/4 can induce both cLDs and nLDs by activating TAG synthesis at the expense of PL synthesis. In contrast, increased fatty acyl chain unsaturation, as seen in Mga2ΔTM cells, only induces cLDs because PL synthesis likely continues concurrently with TAG synthesis.

### Partitioning of PA across the NE influences the site of LD production

The results above suggest that increased lipid unsaturation favors cLD over nLD production by keeping PA levels at the INM critically low. Thus, we reasoned that PA might be shifted across the NE to the ER/ONM. To test this idea, we visualized nuclear and cytoplasmic PA content using the PA sensor with or without the NLS. *ino4*Δ cells, which produce nLDs, have high PA levels at the INM and nLD surface, whereas Mga2ΔTM cells have low PA levels at the INM and few, if any nLDs (Figure 4D). In contrast, the cytoplasmic PA sensor co-localizes with cLDs in both *ino4*Δ and Mga2ΔTM cells, indicating that cLD production may be facilitated by PA in both cases (Figures 4D, S4J, and S4K).

If nLD biogenesis in *mga2ΔTM* cells was suppressed because of low INM PA levels, an increase in INM PA should restore nLD production. To test this critical hypothesis, we assayed nLD formation as well as INM PA levels in the double mutant with deleted *ino4Δ* in addition to *mga2ΔTM*. Importantly, this mutant exhibited increased PA levels at the INM and showed nLD synthesis (Figures 4E, S4H, and S4I). This finding is consistent with the fact that key genes in the PL synthesis branch are downregulated in *ino4*Δ *mga2ΔTM* cells (Figures 4A and S4B). Thus, we suggest that increased lipid unsaturation, which is mimicked by Mga2ΔTM overexpression, triggers increased cLD and decreased nLD production because of an INM PA/PC ratio that is not permissive for nLD formation.

### Lipid unsaturation is highly toxic if not buffered by cLDs

**Figure 5:**
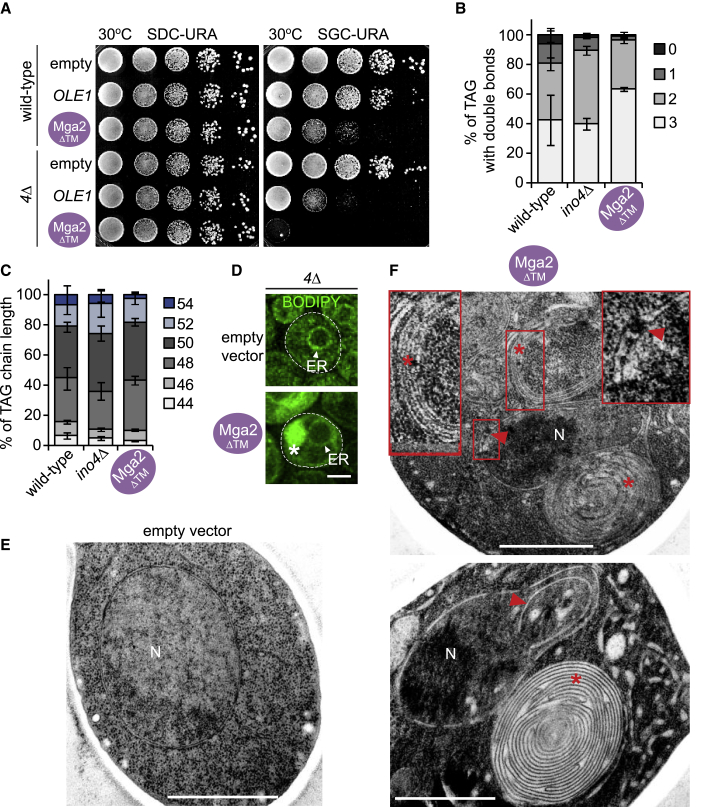
Lipid unsaturation is highly toxic if not buffered by cLDs (A) Growth analysis of wild-type or *4*Δ (*dga1*Δ *lro1*Δ *are1*Δ *are2*Δ) cells transformed with the indicated plasmids. Ole1 and Mga2ΔTM were overexpressed from the galactose-inducible *GAL1* promoter. Growth was followed on SDC-URA (repressed) and SGC-URA (induced) plates. Cells were spotted onto plates in 10-fold serial dilutions and incubated at 30°C. (B) Analysis of TAG fatty acid saturation levels in LDs purified from the indicated strains from 3 biological replicates. Mean value and standard deviation are shown. TAG contains three fatty acyl chains; hence, the number of double bonds can range from 0 to 3. (C) Analysis of TAG fatty acid chain length in LDs purified from the indicated strains from 3 biological replicates. Mean value and standard deviation are depicted. (D) Live imaging of BODIPY-stained *4*Δ cells expressing Mga2ΔTM from the inducible *GAL1* promoter or an empty plasmid. *4*Δ cells are deficient in LDs and BODIPY labels endomembranes instead. Endoplasmic reticulum, ER. Asterisk marks abnormal membrane structure. Scale bar, 2 μm. (E and F) TEM analysis of representative examples of *4Δ* cells overexpressing Mga2ΔTM from the inducible *GAL1* promoter or an empty vector (see also Figure S5). Red asterisk marks membrane stacks/whorls; red arrowhead indicates NE defects including NE expansions and alterations of the perinuclear space. Insets show a magnified view of the marked areas. Nucleus, N. Scale bar, 1 μm.

A key question is why increased lipid unsaturation elicits such a strong cLD response (∼52-fold increase in LD volume in Mga2ΔTM cells compared with wild-type cells). We hypothesized that cLDs sequester UFAs to prevent a deterioration of NE lipid packing and fluidity (Ernst et al., 2016). To test this idea, we raised lipid unsaturation in cells that cannot produce LDs. We overexpressed *OLE1* and *mga2ΔTM* from an inducible *GAL1* promoter in a strain carrying four deletions of genes required for neutral lipid synthesis (“*4Δ* strain”). This yeast strain lacks all four acyltransferases involved in TAG synthesis (i.e., *DGA1*, *LRO1*, *ARE1*, and *ARE2*) and is deficient in LDs (Sandager et al., 2002). While *OLE1* and *mga2ΔTM* overexpression caused a growth defect already in wild-type cells (Figure 5A), these phenotypes were clearly enhanced when LD synthesis was blocked in the *4Δ* strain. The *mga2ΔTM* overexpression became lethal in *4Δ* cells. This suggests that the topological reprogramming of LD synthesis toward cLDs can become essential for coping with lipid unsaturation toxicity.

In line with this idea, we found that LDs isolated from *mga2ΔTM* cells contained a higher percentage of TAG molecules with three double bonds compared with wild-type or *ino4*Δ cells (Figure 5B), whereas the fatty acyl chain length of these TAGs was similar (Figure 5C). To further verify that *4Δ* cells are indeed not capable of producing LDs when challenged with UFAs, we stained them with BODIPY. Notably, we observed large BODIPY-positive structures in the cytoplasm when *mga2ΔTM* was overexpressed in *4Δ* cells (Figure 5D). Ultrastructural analysis revealed that these BODIPY-positive structures likely correspond to tightly curled, onionskin-shaped membrane expansions in the cytoplasm (Figures 5E, 5F, and S5). Thus, the formation of membrane expansions, which likely originate from the ER, could reflect a frustrated attempt of diluting UFAs by increasing the ER surface area when UFA sequestration in cLDs is compromised. Interestingly, we also observed polymorphic aberrations of the NE, including invaginations, herniations or perinuclear blistering. Hence, membrane growth, in contrast to cLD synthesis, is not sufficient to protect the NE from deteriorating. The varied shape changes of the NE are consistent with an increased membrane fluidity due to unsaturated acyl chains. In sum, our data show that sequestration of UFAs in cLDs with a concomitant suppression of nLD production can be physiologically important as it protects the NE from UFA produced membrane destabilization.

### Targeting seipin to the INM is sufficient to produce nLDs

Seipin (yeast Sei1) is a conserved ER protein required for LD biogenesis (Fei et al., 2008; Szymanski et al., 2007). Seipin forms membrane-embedded homooligomeric rings of 11 to 12 subunits with an aperture in the middle (Sui et al., 2018; Yan et al., 2018). Without seipin, cLD and nLD formation is impaired resulting in LDs with aberrant morphologies such as clusters of tiny LDs and/or few “supersized” LDs (Cartwright et al., 2015; Fei et al., 2011; Wang et al., 2016). These defects may arise from abnormal membrane bridges between LDs and ER membranes and thereby an inefficient incorporation of lipids and proteins into nascent LDs (Grippa et al., 2015; Salo et al., 2016). We previously showed that seipin can localize to the INM, where it maintains proper INM-nLD bridges (Romanauska and Köhler, 2018). Notably, seipin binds PA and other anionic PLs, which could influence the curvature and surface tension of the ER and hence LD formation. It is conceivable that seipin utilizes increased PA levels at the INM to regulate nLD formation or is itself capable of concentrating PA at sites of LD formation (Romanauska and Köhler, 2018; Yan et al., 2018).

**Figure 6:**
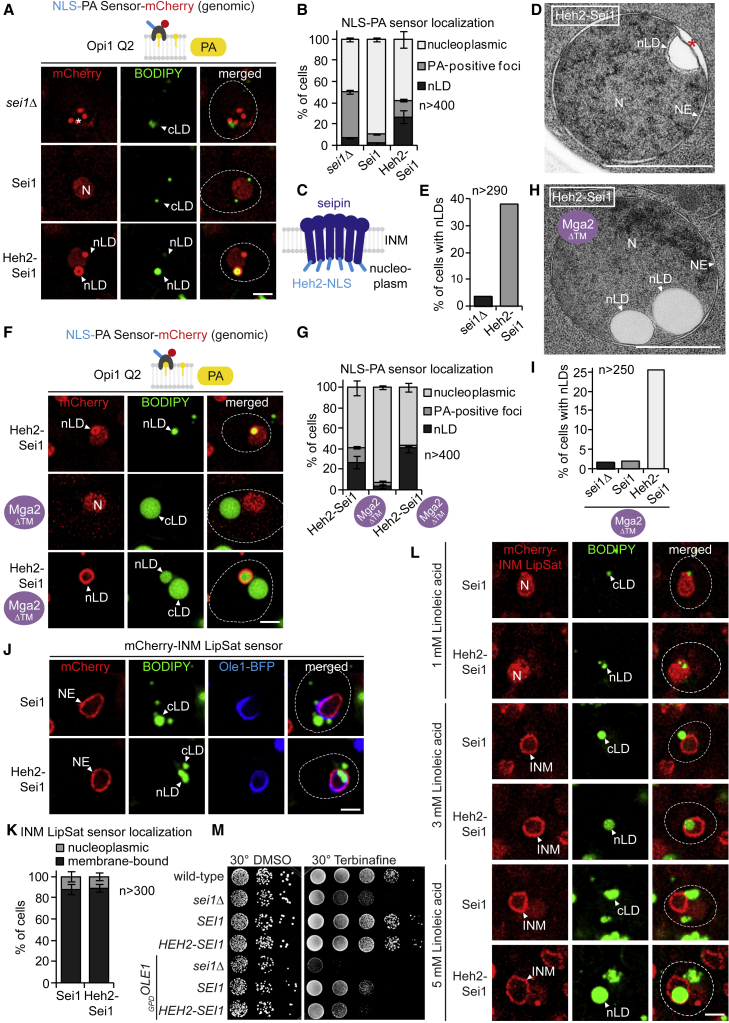
Targeting seipin to the INM is sufficient to produce nLDs (A) Live imaging of *sei1Δ* cells expressing the NLS-PA-mCherry sensor and the indicated *SEI1* constructs or an empty vector (see also Figure S6B). *SEI1* constructs were expressed from the endogenous *SEI1* promoter. nLDs have a BODIPY-positive core surrounded by a PA-rich shell. Nucleus, N; nuclear lipid droplet, nLD. Asterisk marks PA-positive foci. Scale bar, 2 μm. (B) Quantification of NLS-PA-mCherry sensor localization as observed in (A). n = number of analyzed cells obtained from 3 biological replicates. (C) Cartoon of the engineered Sei1 structure with the Heh2-NLS attached. Putative membrane topology is based on cryo-EM models. (D) TEM analysis of a representative example of Heh2-Sei1-expressing cells. Plasmid-based Heh2-Sei1 was expressed from the *SEI1* promoter in a *sei1Δ* strain (see Figures S7A–S7F for a gallery). Nucleus, N; nuclear envelope, NE; nuclear lipid droplet, nLD. Asterisk marks a widened perinuclear space beneath an nLD. Scale bar, 1 μm. (E) Quantification of nLD numbers in (D). n = number of analyzed cells. (F) Live imaging of NLS-PA-mCherry sensor in the indicated strains as a readout for nLD production. nLDs have a BODIPY-positive core surrounded by a PA-rich shell. Nucleus, N; nuclear lipid droplet, nLD. Scale bar, 2 μm. (G) Quantification of NLS-PA-mCherry sensor localization in (F). n = number of analyzed cells obtained from 3 biological replicates. (H) TEM analysis of Heh2-Sei1 expression in Mga2ΔTM cells (see also Figures S7G–S7K for a gallery). Nucleus, N; nuclear envelope, NE; nuclear lipid droplet, nLD. Scale bar, 1 μm. (I) Quantification of nLD numbers in (H). n = number of analyzed cells. (J) Live imaging of an mCherry-tagged INM LipSat sensor in cells that overexpress genomically integrated Ole1-BFP (*GPD* promoter) and contain Sei1 or Heh2-Sei1 constructs. Constructs were transformed into a *sei1Δ mga2Δ* strain. BODIPY stains LDs. Nuclear envelope, NE; nuclear lipid droplet, nLD; cytoplasmic lipid droplet, cLD. Scale bar, 2 μm. (K) Quantification of INM LipSat sensor localization in (J). Phenotypes were classified as membrane bound or nucleoplasmic. Mean value and standard deviation are depicted. n = number of analyzed cells for each condition from 3 biological replicates. (L) Live imaging of an mCherry-tagged INM LipSat sensor co-expressed with Sei1 or Heh2-Sei1 constructs in cells supplemented with the indicated concentration of linoleic acid (dissolved in 1.5% Brij L23 solution). Constructs were transformed into a *sei1*Δ *mga2*Δ strain. BODIPY stains LDs. Nucleus, N; inner nuclear membrane, INM; nuclear lipid droplet, nLD; cytoplasmic lipid droplet, cLD. Scale bar, 2μm. (M) Phenotypic analysis of the indicated strains. Genomically integrated Ole1-BFP was overexpressed from a *GPD* promoter. Growth was followed on SDC-LEU plates with DMSO or supplemented with 100 μg/mL Terbinafine and DMSO. Cells were spotted onto plates in 10-fold serial dilutions and incubated at 30°C.

We wondered whether low seipin activity at the INM plays a role in suppressing nLD formation upon increased lipid unsaturation. To test this hypothesis, we constitutively targeted Sei1 to the INM by appending the NLS of the INM-resident transmembrane protein Heh2 (Figure 6C). Wild-type Sei1 was detected both at the peripheral ER and at the NE (Figure S6A). In contrast, the NLS version of Sei1 (termed Heh2-Sei1) removed the protein from the peripheral ER while targeting the NE, suggesting its nuclear import. Strikingly, the NLS-bearing Sei1 construct induced nLDs in approximately 30% of cells when assessed by fluorescence microscopy (Figures 6A and 6B; see Figure S6B for control). These nLDs had a PA-rich lipid layer on the surface and recruited LD-coating proteins such as the perilipin Pet10 (Figure S6C) as well as the enzyme Dga1 (homologous to human DGAT2) (Choudhary et al., 2020), which is responsible for TAG synthesis (Figure S6D). *sei1Δ* cells displayed clusters of small PA-positive foci in the nucleus in ∼40% of cells (Figures 6A and 6B) (see also Wolinski et al., 2015). However, these PA-positive foci rarely accumulated TAG in their interior, as shown by a lack of colocalization with BODIPY, and they failed to recruit Pet10 and Dga1. The aberrant PA-foci are clearly distinguishable from mature nLDs in Heh2-Sei1 cells. Using TEM, we confirmed nLDs in approximately 40% of *HEH2-SEI1* cells. nLDs were often positioned close to the INM (Figures 6D and 6E, and S7A–S7F) and were located above a widened perinuclear space. These structures likely correspond to the BODIPY-positive and NLS-PA-sensor-enriched structures seen by live-cell fluorescence microscopy.

With this tool in hand, we could test whether nLD formation can be restored in Mga2ΔTM cells, which suppress nLDs in favor of cLDs. Strikingly, expressing Heh2-Sei1 in Mga2ΔTM cells induced nLD formation in ∼40% of cells as shown by the presence of large BODIPY-stainable nLDs with a PA-rich lipid layer on the surface (Figures 6F and 6G), a finding that was confirmed by TEM (Figures 6H, 6I, and S7G–S7K). Heh2-Sei1 also induced nLDs in Ole1-overexpressing cells (Figures S6E and S6F).

These data indicate that nLD formation at the INM results from a synergy between INM PA levels and seipin activity. Given its ability to bind PA, seipin might actively concentrate PA at the base of a nascent LD. If true, induced nLD formation by Heh2-Sei1 should be further enhanced by increased INM PA levels. To test this hypothesis, we expressed Heh2-Sei1 in *opi3Δ* cells, which have elevated INM PA levels (Figures S6G and S6H). Strikingly, high PA levels in conjunction with seipin raised the nLD count from ∼30% to ∼45%. As seipin binds PA, and PA influences the curvature and surface tension of membranes, it is likely that seipin activity is facilitated by increased PA levels at the INM.

Given that LDs can sequester excess UFAs, we asked whether induced nLD formation in Ole1-overexpressing cells can reduce PL unsaturation at the INM. However, the INM LipSat sensor was membrane bound regardless of whether nLDs were formed or not, indicating that INM lipids remained highly unsaturated (Figures 6J and 6K). To exclude the possibility that Ole1-overexpression leads to UFA levels that are too high to be sequestered into nLDs, we determined the lipid unsaturation threshold at which cells fail to clear UFAs from the INM. To this end, we determined the concentration of linoleic acid at which the INM LipSat sensor detects a UFA increase at the INM. Remarkably, this occurred in the narrow range between 1 mM and 3 mM linoleic acid. At 1 mM the INM LipSat sensor was mostly processed and nucleoplasmic, whereas at 3 mM the sensor was mostly unprocessed and INM bound (Figures 6L and S6I). We then asked whether targeting Sei1 to the INM (i.e., Heh2-Sei1), which induces nuclear LDs, would alleviate the UFA burden of the INM via nLD formation and UFA sequestration. Notably, this was not the case despite robust formation of nLDs (Figures 6L and S6I). This suggests that nLD formation cannot efficiently detoxify UFAs at the INM. Even at low doses of a UFA challenge, cells select the cLD route.

Finally, we sought to assess the functional consequences of nLD formation when cells are challenged by high UFA loads. Terbinafine sensitivity was previously used to assess seipin or LD functionality (Choudhary et al., 2015; Hariri et al., 2018; Wang et al., 2014). Terbinafine blocks ergosterol biosynthesis by inhibiting the squalene epoxidase Erg1 (Padyana et al., 2019). As a result, squalene accumulates and becomes lipotoxic when LD biogenesis is defective (Valachovic et al., 2016). To this end, we spotted cells on plates containing Terbinafine, to which *sei1Δ* cells are particularly sensitive (Figure 6M). HEH2-SEI1 cells grew essentially like wild-type *SEI1* suggesting that increased nLD formation is not toxic under these conditions. Deleting *SEI1* in cells that overexpress *OLE1* was highly toxic, consistent with an essential function of LD formation in the detoxification of UFAs. The HEH2-SEI1 construct had a stronger growth defect than wild-type *SEI1* when combined with *OLE1* overexpression. Hence, a parsimonious explanation is that UFA toxicity (i.e., *OLE1* overexpression) and squalene toxicity (Terbinafine) cause synthetic sickness when combined with seipin activity at the INM (i.e., *HEH2-SEI1*). This suggests that redirecting UFA storage into the nucleus is not beneficial for cells. And it reinforces the notion that the topological reprogramming of LD biogenesis toward cLDs is a physiologically important mechanism for detoxifying UFAs.

## Discussion

**Figure 7:**
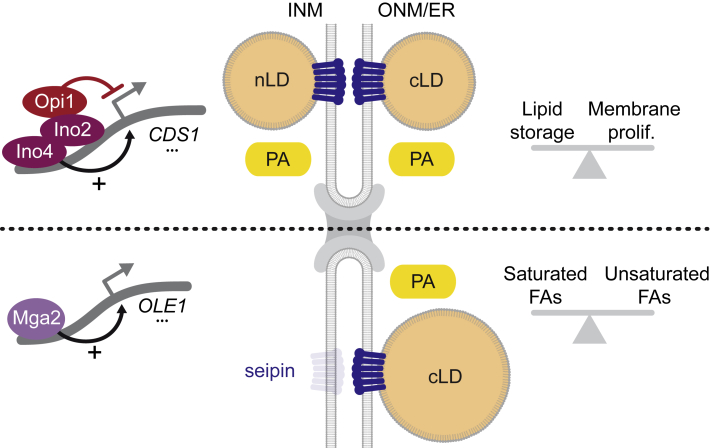
Reprogramming LD biogenesis from the INM to the ONM Model describes how two distinct transcriptional circuits, together with seipin and PA distribution across the NE, collectively regulate the balance of nLD/cLD production in response to UFAs and nutrients. Inhibition of Ino2/4 by Opi1 stimulates both cLD and nLD formation and correlates with high PA levels at the INM and ONM/ER. This pathway globally determines whether cells invest into lipid storage or membrane proliferation. In contrast, the Mga2-Ole1 circuit preferentially induces cLDs, while nLD biogenesis is inhibited, possibly through low PA levels and decreased seipin activity at the INM (transparent icon). The reprogramming of LD biogenesis from the INM to the ONM may protect the nucleus from UFA-induced lipotoxicity.

We have uncovered how the INM recalibrates its lipid saturation after a lipotoxic assault. Despite its ability to produce nLDs, our data suggest that the INM is not always detoxified of UFAs by nLDs, but rather by cLDs formed from the ONM and ER. A distinct rewiring of two transcriptional programs and a synergistic suppression of seipin and PA activity at the INM redirect toxic lipids away from the nuclear interior (Figure 7). The development of compartment-specific LipSat biosensors will enable further research into the INM’s saturation state in diverse genetic perturbations, additional nutrient conditions and across evolution.

### Lipid packing and viscosity of the INM

We aimed to determine whether and how the INM controls the balance between saturated and unsaturated FAs to maintain adequate lipid packing and viscosity. These features are of general importance for any membrane, since they affect the folding, structure, function, and diffusion of all membrane-embedded proteins (Ernst et al., 2016). They might be of particular importance for the INM, since this territory is topologically a cul-de-sac of the ER, which harbors a specialized chromatin-associated proteome and quality control mechanisms distinct from the ONM/ER (Ungricht and Kutay, 2017). The lipid packing and viscosity of the INM will likely affect numerous aspects of nuclear function including the NE’s shape, size, spacing between INM and ONM, the NE’s resilience to mechanical stress, and possibly even chromatin activity via INM-embedded proteins. INM viscosity may further impact the mechanics of nuclear pore membrane formation during interphase, which proceeds by an inside-out extrusion of the INM (Otsuka et al., 2016), as well as the pathological rupture and re-sealing of the NE in human cancer cells (Hatch et al., 2013), which often display aberrant lipid saturation profiles (Vriens et al., 2019). A key finding of our study is that the INM experiences changes in lipid saturation, which necessitates mechanisms to recalibrate its lipid packing and viscosity.

### cLD production as a specific mechanism for nuclear UFA detoxification

Yeast has no reported enzyme to reverse the activity of Ole1 and increase saturation, necessitating other means of UFA detoxification. In the absence of such enzyme, cells rely on fat storage organelles to discard UFAs. LD formation has previously been suggested to promote ER homeostasis, as the addition of an excess of dietary UFAs correlates with more cellular LDs. LD-deficient cells accumulate FAs within ER membranes, resulting in growth sensitivity when cultured with nutrient UFAs (Garbarino et al., 2009). However, the pleiotropic effects of nutrient overload experiments have complicated a mechanistic analysis of how acyl chain saturation specifically contributes to this toxicity.

Here, we managed to alter Ole1 activity or the Mga2 transcription circuit without relying on exogenous FA supplementation. This allowed us to specifically examine the response to unsaturation. Our earlier work had shown that the INM is capable of nLD formation, which is triggered by mechanisms that increase PA levels at the INM and draw PA into the storage branch (Romanauska and Köhler, 2018). nLD and cLD formation were co-regulated and governed by the amount of Opi1 inside the nucleus, where Opi1 represses the promoters of genes involved in PL synthesis. Given that Ole1 activity induces LDs, we expected that INM UFAs would be detoxified specifically by nLDs, which are connected to the INM by membrane bridges. Surprisingly, we found that excess UFAs strongly stimulated cLD formation, whereas nLD synthesis was suppressed. Interestingly, oleic acid (C18:1) produces both nLDs and cLDs; however, linoleic acid (C18:2) induced only cLDs. Thus, a second double bond in a C18 carbon chain may already necessitate a shift to cLD production.

Why are nLDs not used as a major route to detoxify UFAs? First, a full-fledged UFA response (e.g., Mga2ΔTM cells) causes a massive increase in cytoplasmic LD content. Flooding the nucleus with nLDs may interfere with genome function, causing compression and displacement of chromatin and impeding nucleocytoplasmic transport. Thus, nLDs may not be “inferior” per se in packaging UFAs, however, the genome may not permit UFA disposal in its neighborhood. Second, nLDs interact with transcription factors, which likely is one of their main regulatory roles. Opi1, the master switch of lipid metabolism in yeast, is recruited to nLDs (Romanauska and Köhler, 2018), thereby controlling Opi1 transcriptional activity. Conceivably, an excessive increase in nLD surface area may perturb the balance of LD-bound and unbound transcription factors and interfere with gene regulation. Third, excess nLDs may sequester histones or other nuclear proteins, which is one of their natural roles in *Drosophila* embryonic development (Li et al., 2012), but would be detrimental in a somatic nucleus. Finally, it is possible that the breakdown of UFA-charged LDs by lipases creates by-products that are toxic in a nuclear environment (Rockenfeller et al., 2018). In sum, as with fat storage diseases in general, LDs may be harmful when present in excess or in the wrong cellular compartment.

### Reprogramming LD biogenesis from the INM to the ONM

How the sites of LD biogenesis are spatially determined within the vast ER network is a key question. Although the frequency of nLDs in yeast cells is generally lower than cLDs, the machinery for LD biogenesis is present at the INM. Hence, cells can decide between three interconnected territories (i.e., INM, ONM, and ER) for where to make LDs and this should impact the flux of metabolites and the functional outcome of TAG synthesis. Candidate factors for defining the sites of LD formation include assembly proteins such as seipin, non-bilayer lipids like PA, enzyme location, membrane surface tension, and inter-organelle contacts (Henne et al., 2020). Seipin is recruited to nascent LDs as they mature and is among the first proteins observed at neutral lipid “lens”-like structures between the leaflets of the membrane bilayer. In this complex scenario, tools are needed to define which factors are necessary and sufficient to spatially define LD biogenesis.

A situation of acute lipotoxic stress gave us unique insights into how cells define whether to make LDs from the INM or ONM/ER. We suggest that the two major contributing factors in this decision-making are seipin activity and the availability of PA at the INM versus ONM/ER. Importantly, nLD formation in UFA-challenged cells could be restored either by genetically increasing PA levels at the INM or by targeting Sei1 to the INM. How seipin activity and PA levels at the INM are regulated will be an important subject of future investigations. In principle, this could occur by altering subcellular Sei1 localization via NPC transport or by regulating the activity of INM-resident Sei1. PA, which diffuses laterally within the INM-ONM-ER network, may be altered in its concentration either by local synthesis or by local consumption. Interestingly, PA was reported to bind human seipin *in vitro* (Yan et al., 2018). Hence, seipin may play an active role in concentrating PA at sites of LD formation. In summary, we propose that a metabolic rewiring endows the INM/ONM/ER network with an adaptive plasticity fundamental to lipotoxic resistance and cell survival under ever-changing environmental conditions.

In humans, adipose tissue is essential for buffering lipid excess by sequestering FAs into TAGs, thereby protecting the body from lipotoxicity. Various conditions result in supraphysiological TAG accumulation in the liver. Obesity-related steatosis is the hepatic manifestation of the metabolic syndrome and represents a major public health problem (Gluchowski et al., 2017). Interestingly, hepatocytes can package TAG into at least three kinds of lipid stores—cLDs, nLDs, and very-low-density lipoprotein (VLDL) particles (Soltysik et al., 2019). Our study revealed a situation where lipid storage is directed away from the nucleus, potentially to protect the nucleus from adverse effects. In contrast, the massive nLD accumulation that was recently described in a mouse model of nonalcoholic fatty liver disease (NAFLD; reported to affect ∼34% of the US population) could be an example of a disease-related nLD/cLD imbalance (Shin et al., 2019). It will be interesting to explore whether nLD formation in hepatic statosis causes nuclear lipotoxicity, whether compartment-specific imbalances of hepatocyte LD metabolism contribute to liver disease and whether NE metabolism can be pharmacologically reprogrammed.

### Limitations of the study

Key aspects of lipid metabolism are conserved between budding yeast and metazoa. Future studies need to address how the reprogramming of lipid metabolism across the NE occurs in different organisms and cell types, where variations of this theme can be expected. We report that seipin and PA are critical factors for LD formation at the INM. Other yet unknown factors likely contribute to this process and remain to be identified. This also includes the mechanism by which nLD biogenesis factors are targeted to the INM via NPCs.

## STAR★Methods

### Key resources table

**Table:** 

REAGENT or RESOURCE	SOURCE	IDENTIFIER
**Antibodies**		

Mouse monoclonal anti-mCherry	Abcam	Cat.#ab125096; RRID: AB_11133266
Mouse monoclonal anti-GFP (clones 7.1 and 13.1)	Roche	Cat.#ab11814460001; RRID: AB_390913
Mouse monoclonal anti-Pgk1	Abcam	Cat.#ab113687; RRID: AB_10861977
Peroxidase AffiniPure Goat anti-Mouse IgG	Jackson ImmunoResearch	Cat.#115035008; RRID: AB_2313585

**Chemicals, peptides, and recombinant proteins**		

BODIPY™ 493/503	Thermo Fisher Scientific	Cat.# D3922
Zymolyase® -20T	AMSBIO	Cat.# 120491-1
Oleic acid	Sigma-Aldrich	Cat.# O1008
Linoleic acid	Sigma-Aldrich	Cat.# L1376
Stearic acid	Sigma-Aldrich	Cat.# S4751
Brij® L23 solution	Sigma-Aldrich	Cat.# B4184
Terbinafine hydrochloride	Sigma-Aldrich	Cat.# T8826

**Critical commercial assays**		

Ribo-Zero Gold rRNA Removal Kit (Yeast)	Illumina	Cat.# MRZY1324
NEBNext® Ultra™ II Directional RNA Library Prep Kit	NEB	Cat.# E7760L

**Deposited data**		

RNA-Seq data	This paper	GEO: GSE156951

**Experimental models: Organisms/strains**		

See Table S1		N/A

**Recombinant DNA**		

See Table S2		N/A

**Software and algorithms**		

ImageJ	NIH	https://imagej.nih.gov/ij/
Fiji	NIH	https://fiji.sc/
GraphPad Prism	GraphPad	http://www.graphpad.com/
softWoRX	GE Healthcare	N/A
STAR	(Dobin et al., 2013)	https://github.com/alexdobin/STAR/releases
HTSeq	(Anders et al., 2015)	https://htseq.readthedocs.io/en/master/
R	R Core Team	https://www.r-project.org/

### Resource availability

#### Lead contact

Further information and requests for resources and reagents should be directed to and will be fulfilled by the Lead Contact, Dr. Alwin Köhler (alwin.koehler@maxperutzlabs.ac.at).

#### Materials availability

All plasmids and strains generated in this study are available upon request from the Lead Contact.

### Experimental model and subject details

#### Strains and media

All yeast strains and plasmids used in this study are listed in Table S1 and S2, respectively. Genes in yeast were tagged/deleted by a standard one-step PCR-based technique. Microbiological techniques followed standard procedures. Cells were grown in standard yeast extract peptone dextrose (YPD) prior transformation or in SDC+All for experiments, or when transformed with plasmids in selective synthetic dextrose complete (SDC) drop-out media at 30°C. To induce protein production from the *GAL1* promoter, cells were grown in media containing 2% raffinose before adding galactose to a final concentration of 2% for 6 hrs. Fatty acid-containing growth media were prepared from standard SDC media containing 0.1% glucose, the indicated concentration of fatty acids (stearic, oleic or linoleic acid, all from Sigma-Aldrich) and 1.5% Brij L23 solution (Sigma-Aldrich). Fatty acids were omitted from the control medium.

### Method details

#### Live-cell imaging of yeast

Exponentially growing cells (unless indicated otherwise) were immobilized on microscope slides with agarose pads and imaged on a DeltaVision Elite microscope (GE Healthcare). Images were acquired with a 60x oil immersion objective and recorded with a CoolSNAP HQ2 CCD camera (Photometrics). Deconvolution was carried out using softWoRx software (GE Healthcare). Images were processed with ImageJ. Cell contours were marked with a dashed white line based on brightfield imaging. To stain lipid droplets, BODIPY™ 493/503 (final concentration 5.7 μM, Thermo Fisher Scientific) was added and cells were imaged after 20 min.

#### Yeast growth assay

For dot-spot assays, cells were grown exponentially, harvested and resuspended to a final OD_600_ of 0.5. 10-fold serial dilutions were prepared, spotted on appropriate plates and incubated at 30°C. For Terbinafine plates, 100 μg/mL Terbinafine hydrochloride (Sigma-Aldrich) dissolved in DMSO was used. For Terbinafine control plates, only DMSO was added to the media.

#### Transmission electron microscopy (TEM)

*ino4Δ* and Mga2*Δ*TM, and *sei1Δ* cells were grown at 30°C in synthetic media, while *4Δ* and *4Δ* Mga2*Δ*TM (*GAL1* promoter) cells were grown in media containing 2% galactose for 6 hours prior to TEM analysis. Pelleted cells were mixed 1:1 with 10% BSA, used as a filler, and transferred into the 100 μm cavity of a 3 mm aluminum specimen carrier. This carrier was sandwiched with a flat 3 mm aluminum carrier and immediately high pressure frozen in a HPF Compact 01 (both carriers and high pressure freezer from Engineering Office M. Wohlwend GmbH). The frozen samples were subsequently transferred into a Leica EM AFS-2 freeze substitution unit (Leica Microsystems). Over a period of 4 days, samples were substituted in a medium of acetone containing 2% osmium tetroxide (Agar Scientific), 0.2% uranyl acetate and 5% water. Freeze substitution was performed according to the following protocol: 40 hrs at –90°C, warm up at a rate of 2°C per hour to –54°C, 8 hrs at –54°C, warm up at a rate of 5°C per hour to –24, 15 hrs at –24°C, warm up at a rate of 5°C per hour to 0°C, 2 hrs at 0°C. At 0°C samples were taken out and washed 3 times in anhydrous acetone (on ice) and infiltrated with Agar 100 Epoxy resin (Agar Scientific), in a graded series of acetone and resin over a period of 3 days. Polymerization took place at 60°C. Ultra-thin sections with a nominal thickness of 70 nm were cut using a Leica UCT ultramicrotome (Leica Microsystems). Examination regions on the sections were randomly selected and inspected with a FEI Morgagni 268D (FEI) operated at 80 kV. Digital images were acquired using an 11 megapixel Morada CCD camera (Olympus-SIS).

#### Lipid droplet isolation and mass spectrometry

Exponentially growing yeast cultures were harvested and washed with water and Zymolase buffer (1 M Sorbitol; 50 mM Tris-HCl, pH=7.5; 10 mM MgCl_2_). Cells were incubated for 15 min at 30°C with shaking and centrifuged for 5 min 4000 rpm. Cell pellets were resuspended in 30 mL Zymolase buffer and 20 mL YPD/S (YPD; 1 M Sorbitol). 20T Zymolase (AMSBIO) was added and incubated for 1.5 hours at 30°C with shaking. After spheroplasting, cold 1 M Sorbitol was added and cells were pelleted at 4000 rpm for 10 min. The pellet was resuspended in lysis buffer (8% PVP-40; 20 mM K-phosphate, pH=6.5; 7.5 μM MgCl_2_; 5 mM DTT) and PMSF and protease inhibitors were added immediately before lysis. Spheroroplasts were homogenized using a Polytron homogenizer (Kinematica, Switzerland). Lysate was loaded on top of a sucrose gradient (8 mL 2.50 M sucrose; 10 mM Bis-Tris-HCl pH=6.5; 0.1 mM MgCl_2_ / 1.875 M sucrose; 10 mM Bis-Tris-HCl pH=6.5; 0.1 mM MgCl_2_ containing PMSF and protease inhibitors). Gradients were centrifuged at 28,000 rpm (103,745 g) for 25 min at 4°C. The upper lipid droplet fraction was loaded on top of the cushion buffer (20 mM Hepes-KOH pH 7.4; 100 mM KCl; 2 mM MgCl_2_ with protease inhibitors). Centrifugation was performed at 120,000 g for 1 h at 4°C. The upper lipid droplet fraction was removed and frozen in N_2_.

Lipids were extracted using liquid-liquid extraction, employing a mixture of 300 μl chloroform, 150 μl methanol and 100 μl acidified (0.2% formic acid in water) 0.25 M NaCl. The lower phase was transferred to a new Eppendorf tube and 100 μl acidified 0.25 M NaCl (0.2% formic acid in water) was added. The lower phase was transferred to an HPLC glass vial, mixed with an equal amount of methanol and analyzed by LC-MS.

Lipids were quantified by injecting 1 μl of the extract on an Ultimate 3000 RSLC (Thermo Fisher Scientific) that is directly coupled to a TSQ Vantage mass spectrometer (Thermo Fisher Scientific). For separation, a Kinetex C8 column was used (100 Å, 150 x 2.1 mm), employing a flow rate of 100 μl/min. A 7-minute-long linear gradient was used from 90% A (60% acetonitrile, 0.4% formic acid, 39.6% water, 10 mM ammonium acetate) to 95% B (90% isopropanol, 9.6% acetonitrile, 0.4% formic acid, 10 mM ammonium acetate). TAG was analyzed in the positive ion mode and quantified by measuring the ammonium adducts in single MS mode. PA was analyzed in the negative ion mode, utilizing the neutral loss of the fatty acids from the respective precursor ion. Retention times of TAG and PA were calibrated by analyzing standard mixtures. For whole cell analysis, the same amount of cells were used based on optical density (OD) measurements.

#### Total RNA isolation

Total RNA was extracted from 12 mL of an exponentially growing culture using a hot phenol method. Briefly, cells were washed with water and the cell pellet was resuspended in 500 μL AE buffer (50 mM sodium acetate pH=5.2; 10 mM EDTA). 100 μL 10% SDS and 500 μL acidic phenol were added to the samples and incubated for 10 min at 65°C. Samples were then cooled on ice and centrifuged for 15 min at 17g at 4°C. The upper phase was mixed with 500 μL chloroform and centrifuged for 10 min at 17g at room temperature. The upper phase was mixed with 1/10 volume of 3M sodium acetate and an equal volume of isopropanol. After centrifugation for 45 min at 17g at room temperature, the pellet was washed with 70% ethanol. After centrifugation, ethanol was removed and the pellet was air-dried. The pellet was resuspended in RNase-free water for digestion with DNase.

#### Transcriptome analysis

For RNA sequencing, total RNA was depleted from ribosomal RNA and then reverse transcribed using random oligomer primers (Ribo-Zero, Illumina). Sequencing was performed on an Illumina platform (HiSeq2500 instrument using single-end sequencing with 50bp read length). The reads were mapped to the *Saccharomyces cerevisiae* Ensembl R64-1-1 reference genome with STAR (version 2.5.1b) (Dobin et al., 2013). The read counts for each gene were detected using HTSeq (version 0.5.4p3) (Anders et al., 2015). The counts were normalised using the TMM normalization from edgeR package in R. Prior to statistical testing the data was voom transformed. The differential expression between the sample groups was calculated with limma package in R.

#### Immunoblotting

Yeast whole-cell extracts were prepared, normalized for protein concentration and analyzed by western blotting according to standard procedures. Antibodies were used according to the manufacturer’s instructions: mouse monoclonal anti-mCherry (Abcam); mouse monoclonal anti-GFP (clones 7.1 and 13.1) (Roche); mouse monoclonal anti-Pgk1 (Abcam); peroxidase AffiniPure Goat anti-Mouse IgG (Jackson ImmunoResearch).

### Quantification and statistical analysis

Data are depicted as mean with standard deviation. n values and biological replicates are indicated in the figure legends. Data normality was determined using the Shapiro-Wilk test. Statistical significance was evaluated by t-test or Mann-Whitney test depending on data normality using the GraphPad Prism software. Statistical significance is indicated in figures or figure legends (^∗^ represents p<0.05, ^∗∗^ p<0.01, ^∗∗∗^ p<0.001). Detailed descriptions of phenotype quantification are provided below.

To quantify the percentage of cells with nLDs, the following criteria were used: nLDs are defined as BODIPY-positive structures present within the confines of a Heh2-mCherry-labeled INM.

To quantify the NLS-PA sensor localization, the following four criteria were used: “nLDs” are defined as spherical, BODIPY-positive structures surrounded by the fluorescent NLS-PA sensor. “INM localization” is defined as a fluorescent labeling of the NE in the absence of nLDs with a peak fluorescence intensity at least 1.3 times higher than the nucleoplasm. "PA-positive foci" are defined as NLS-PA sensor-labelled foci in the nucleoplasm that did not co-localize with BODIPY. When none of the above mentioned criteria were met, the sensor was classified as “nucleoplasmic".

Pct1-mCherry localization was defined as “membrane-bound” if the peak fluorescent intensity of the NE was at least 1.3 times higher than the nucleoplasm; otherwise it was classified as "nucleoplasmic".

Automated quantification of cellular LD volume was performed using the Fiji plugin "Trainable Weka Segmentation". The segmentation classifier was trained with two classes - to recognize LDs (class 1) and to recognize the background (class 2). The "Watershed" plugin was used to separate adjacent LDs. Next, particle analysis was performed and the volume of each LD was quantified. To calculate LD volume per cell, the sum of individual LD volumes was divided by cell number.

For EM analysis, the presence of nLDs and INM evaginations was scored. Only cells with nuclei exhibiting a diameter ≥ 1 μm were counted.

INM/ER LipidSat sensor localization was defined as “membrane-bound” if the peak fluorescent intensity of the NE was at least 1.3 times higher than the fluorescence of the nucleoplasm; otherwise it was classified as "nucleoplasmic". For the analysis of INM/ER LipSat sensor processing, the sum of the total Mga2 protein was calculated from the immunoblots and the percentage of p120^∗^ and p90^∗^ or Heh2-p120^∗^ and Heh2-p90^∗^ relative to the total amount was determined. The intensity of the bands was measured in ImageJ.

## Data Availability

The RNA-Seq data used for analysis are deposited in the GEO repository under accession code GSE156951 and are publicly available as of the date of publication. This paper does not report any original code. Any additional information required to reanalyze the data reported in this paper is available from the Lead Contact upon request.

## Supporting citations

The following references appear in the supplemental information: Hofbauer et al. (2018); Loewen et al. (2004); Sikorski and Hieter (1989).

## Supplemental information

Document S1. Figures S1–S7 and Tables S1 and S2

Document S2. Article plus supplemental information
