# Supplementary material for: Reprogrammed lipid metabolism protects inner nuclear membrane against unsaturated fat
Source: Dev Cell. 2021 Sep 27;56(18):2562–2578.e3. doi: 10.1016/j.devcel.2021.07.018 (PMC8480995; doi:10.1016/j.devcel.2021.07.018)
Supplement: Document S1. Figures S1–S7 and Tables S1 and S2 [file mmc1.pdf]

Developmental Cell, Volume *56*

Supplemental information

Reprogrammed lipid metabolism protects inner nuclear membrane against unsaturated fat

Anete Romanauska and Alwin Köhler

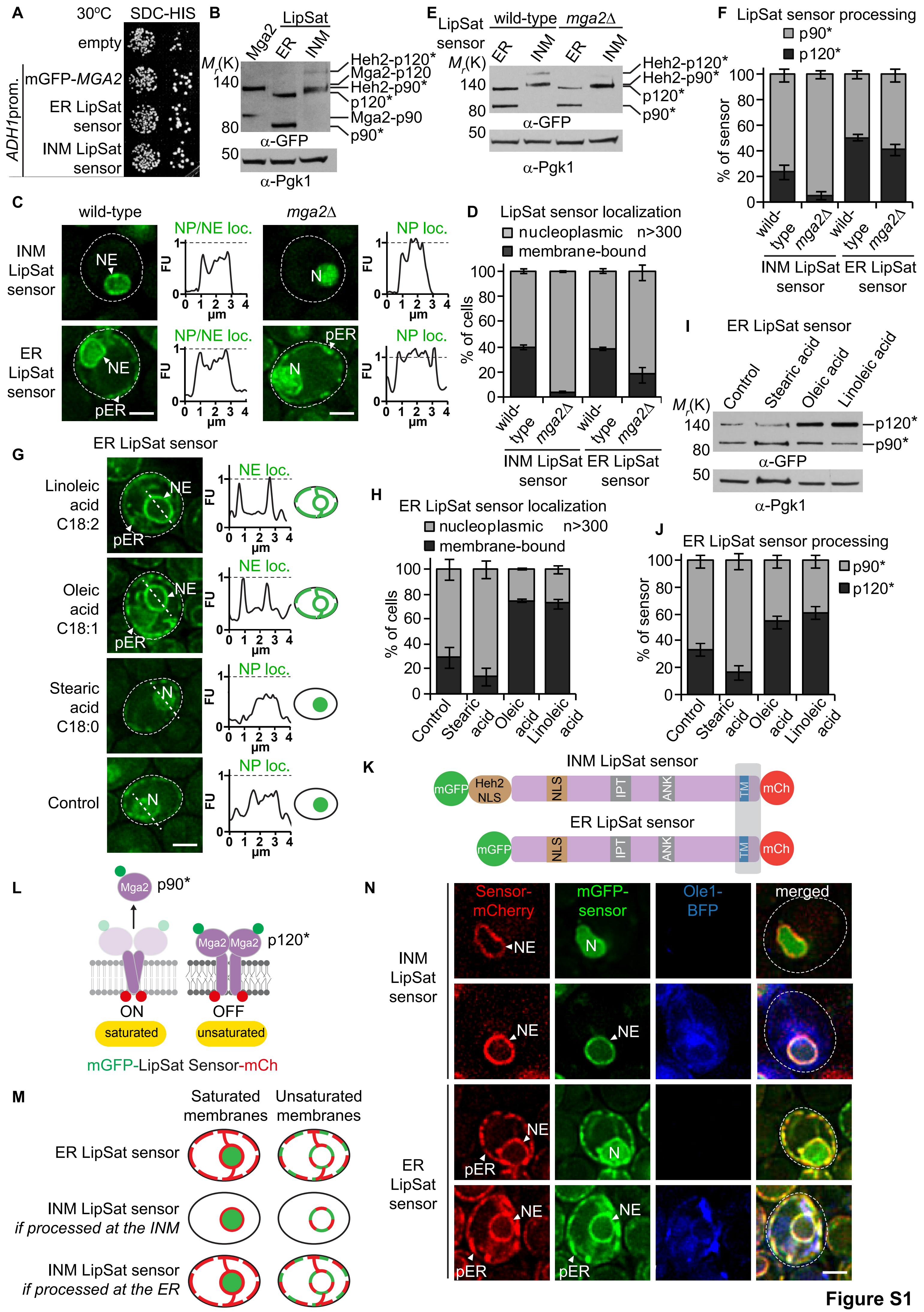
A
30ºC SDC-HIS
empty
mGFP-MGA2
ER LipSat sensor
INM LipSat sensor
ADH1prom.
B
LipSat
Mga2 ER INM
Mr(K)
140
80
50
α-GFP
α-Pgk1
Heh2-p120*
Mga2-p120
Heh2-p90*
p120*
Mga2-p90
p90*
E
LipSat sensor
wild-type mga2Δ
ER INM ER INM
α-GFP
α-Pgk1
F
LipSat sensor processing
p90*
p120*
% of sensor
wild-type mga2Δ wild-type mga2Δ
INM LipSat sensor
ER LipSat sensor
C
wild-type
mga2Δ
INM LipSat sensor
ER LipSat sensor
NP/NE loc.
NP loc.
FU
µm
NE
N
pER
D
LipSat sensor localization
nucleoplasmic n>300
membrane-bound
% of cells
I
ER LipSat sensor
Control
Stearic acid
Oleic acid
Linoleic acid
G
ER LipSat sensor
Linoleic acid C18:2
Oleic acid C18:1
Stearic acid C18:0
Control
NE loc.
H
ER LipSat sensor localization
J
ER LipSat sensor processing
K
INM LipSat sensor
mGFP
Heh2 NLS
NLS
IPT
ANK
TM
mCh
ER LipSat sensor
L
Mga2 p90*
Mga2 Mga2 p120*
ON
OFF
saturated
unsaturated
mGFP-LipSat Sensor-mCh
M
Saturated membranes
Unsaturated membranes
ER LipSat sensor
INM LipSat sensor if processed at the INM
INM LipSat sensor if processed at the ER
N
Sensor-mCherry
mGFP-sensor
Ole1-BFP
merged
INM LipSat sensor
ER LipSat sensor

Figure S1

Supplementary Figure 1. Characterization of LipSat sensor activity in response to nutrients and genetic perturbations, Related to Figures 1 and 2.

(A) Lipid Saturation (LipSat) sensors, expressed in *mga2Δ* cells, do not impair cell growth. Phenotypic analysis of plasmid-based full-length *MGA2* (wild-type control) and the indicated Lipid Saturation (LipSat) sensors. Growth was followed on SDC-HIS plates. Cells were spotted onto plates in 10-fold serial dilutions and incubated at 30°C.

(B) Protein levels of wild-type Mga2 (expressed from endogenous *MGA2* promoter) compared to the ER and INM LipSat sensors, both expressed from the *ADH1* promoter. Pgk1 serves as a loading control.

(C) Comparison of wild-type and *mga2Δ* cells expressing the indicated LipSat sensors. Sensor fluorescence intensity was quantified across a line spanning the nucleus. For comparison the FU value 1 is marked with a horizontal dashed line. LipSat sensor expression in wild-type cells might lead to some heterodimerization with endogenous Mga2, which is excluded in the *mga2Δ* background. Arbitrary Fluorescence Units, FU; nucleus, N; peripheral endoplasmic reticulum, pER; nuclear envelope, NE; nucleoplasmic localization, NP loc.; nuclear envelope localization, NE loc. Scale bar, 2 μm.

(D) Quantification of LipSat sensor localization in **(C)**. Phenotypes were classified as membrane-bound or nucleoplasmic. Mean value and standard deviation are depicted. n = number of analyzed cells for each condition from 3 biological replicates.

(E) Immunoblotting analysis of LipSat sensor processing. Samples were taken from cell cultures used in **(C)**. GFP-tagged Heh2-p120*/p90* fragments have a higher molecular weight than p120/p90. Pgk1 serves as a loading control.

(F) Quantification of LipSat sensor processing in **(E)**. The percentage of Heh2-p120* and Heh2-p90* or p120* and p90* relative to total amount of sensor was quantified. The mean value and standard deviation from 3 biological replicates are depicted. **(G)** Live imaging of *mga2Δ* cells expressing the plasmid-based ER LipSat sensor supplemented with the indicated fatty acids (16 mM). Sensor fluorescence intensity was quantified across a line spanning the nucleus. For comparison the FU value 1 is marked with a horizontal dashed line. Dashed white line indicates the cell contour. Arbitrary Fluorescence Units, FU; nucleus, N; peripheral endoplasmic reticulum, pER; nuclear envelope, NE; nucleoplasmic localization, NP loc.; nuclear envelope localization, NE loc. Scale bar, 2 μm.

(H) Quantification of ER LipSat sensor localization in **(G)**. Phenotypes were classified as: membrane-bound or nucleoplasmic. Mean value and standard deviation are depicted. n = number of analyzed cells for each condition from 3 biological replicates.
(I) Immunoblotting analysis of ER LipSat sensor processing in **(G)**. Pgk1 serves as a loading control.
(J) Quantification of ER LipSat sensor processing in **(I)**. The percentage of p120* and p90* relative to total amount of sensor was quantified. The mean value and standard deviation from 3 biological replicates are depicted.
(K) Cartoon of ER LipSat sensor and INM LipSat sensor carrying an N-terminal mGFP tag and a C-terminal mCherry tag.
(L) Probing the compartment of LipSat processing. When saturated lipids increase (ON), the mGFP-tagged portion of the sensor is processed, released from the membrane and driven into the nucleus by its cryptic NLS. In contrast, the mCherry-tagged transmembrane (TM) part remains in the same membrane location. In the OFF state (unsaturated lipids), the sensor is not processed and the mGFP and mCherry signals co-localize.
(M) Cartoon of possible LipSat sensor processing sites in the cell. If the INM sensor was processed at the INM, the mCherry-tagged TM fragment should always localize to the INM. If INM sensor processing occurred in the ER, the mCherry-tagged TM fragment should remain in the ER.
(N) Live imaging of LipSat sensors carrying an N-terminal mGFP and a C-terminal mCherry tag in *mga2*Δ cells. Ole1-BFP was overexpressed to increase UFA levels. Note that mCherry and mGFP fluorescence of the INM LipSat sensor co-localize at the INM consistent with UFA sensing and processing inside the nucleus. Processing of the LipSat sensor occurs on the N-terminal, nucleoplasmic side of the transmembrane helix (TM). The mCherry tag, however, is located on the C-terminal side of the TM in the perinuclear space. Hence, after processing, the mGFP part is released into the nucleoplasm, whereas the mCherry remains attached to the INM-bound TM. Nucleus, N; peripheral endoplasmic reticulum, pER; nuclear envelope, NE. Scale bar, 2 μm.

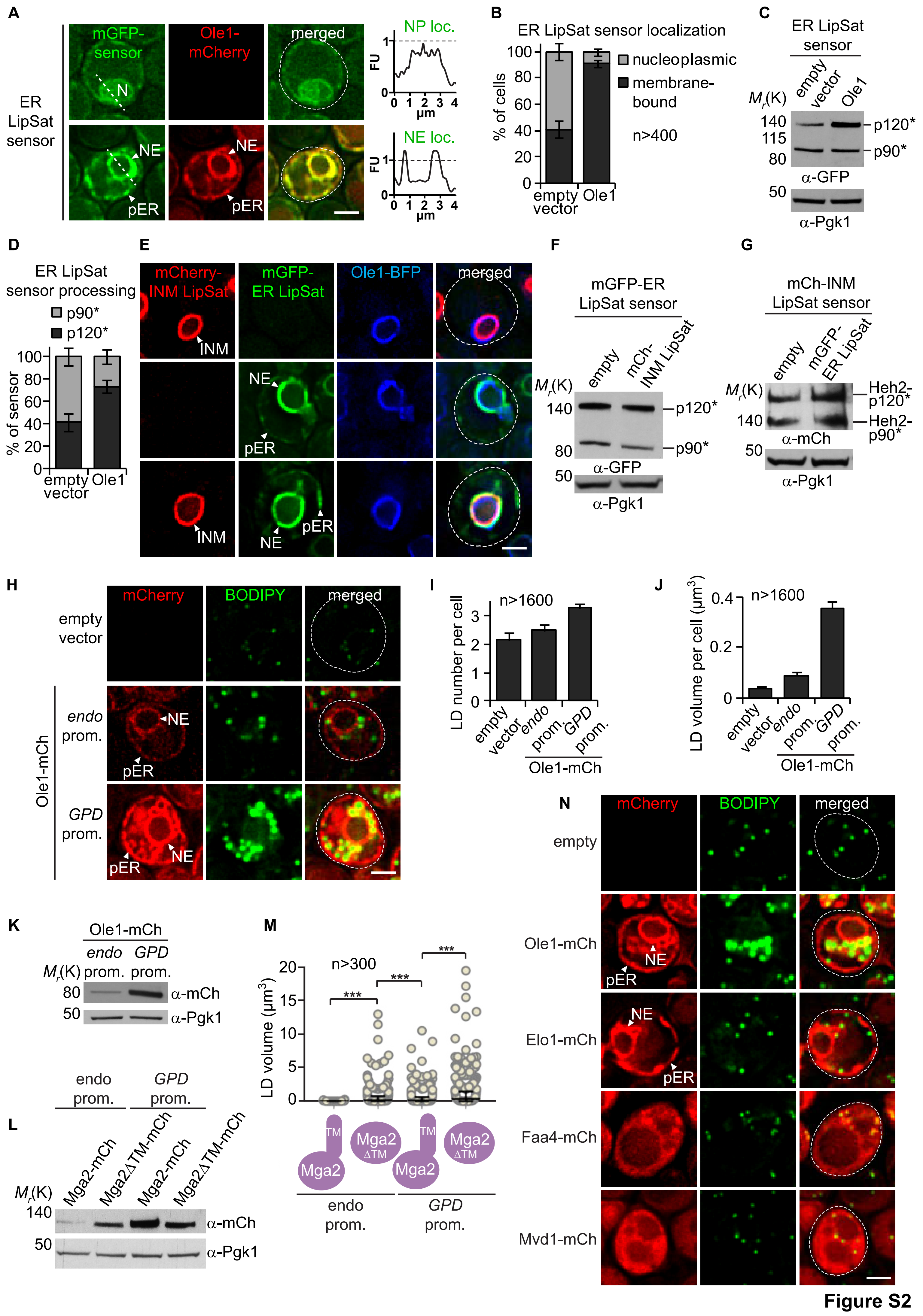
A
ER LipSat sensor
mGFP-sensor
Ole1-mCherry
merged
N
NE
pER
NP loc.
NE loc.
FU
µm
B
ER LipSat sensor localization
% of cells
nucleoplasmic
membrane-bound
n>400
empty vector
Ole1
C
ER LipSat sensor
empty vector
Ole1
M_r(K)
140
115
80
50
p120*
p90*
α-GFP
α-Pgk1
D
ER LipSat sensor processing
p90*
p120*
% of sensor
empty vector
Ole1
E
mCherry-INM LipSat
mGFP-ER LipSat
Ole1-BFP
merged
INM
NE
pER
F
mGFP-ER LipSat sensor
empty
mCh-INM LipSat
p120*
p90*
α-GFP
α-Pgk1
G
mCh-INM LipSat sensor
empty
mGFP-ER LipSat
Heh2-p120*
Heh2-p90*
α-mCh
α-Pgk1
H
mCherry
BODIPY
merged
empty vector
Ole1-mCh
endo prom.
GPD prom.
I
n>1600
LD number per cell
empty vector
endo prom.
GPD prom.
Ole1-mCh
J
n>1600
LD volume per cell (µm³)
K
Ole1-mCh
endo prom.
GPD prom.
80
50
α-mCh
α-Pgk1
L
endo prom.
GPD prom.
Mga2-mCh
Mga2ΔTM-mCh
α-mCh
α-Pgk1
M
n>300
LD volume (µm³)
***
TM
Mga2
Mga2 ΔTM
N
empty
Ole1-mCh
Elo1-mCh
Faa4-mCh
Mvd1-mCh
Figure S2

Supplementary Figure 2. Ole1 regulates lipid droplet formation, Related to Figures 2 and 3.

(A) Live imaging of cells expressing the ER LipSat sensor together with Ole1-mCherry (bottom panel) or an empty vector (top panel). Ole1-mCherry was expressed from the strong *GPD (TDH3)* promoter. Plasmids were transformed into *mga2*Δ cells. Sensor fluorescence intensity was quantified across a line spanning the nucleus. For comparison the FU value 1 is marked with a horizontal dashed line. Arbitrary Fluorescence Units, FU; nucleus, N; peripheral endoplasmic reticulum, pER; nuclear envelope, NE; nucleoplasmic localization, NP loc; nuclear envelope localization, NE loc. Scale bar, 2 μm.

(B) Quantification of ER LipSat sensor localization in **(A)**. Phenotypes were classified as membrane-bound or nucleoplasmic. Mean value and standard deviation depicted. n = number of analyzed cells for each condition from 3 biological replicates.

(C) Immunoblotting analysis of ER LipSat sensor processing in **(A)**. Pgk1 serves as a loading control.

(D) Quantification of ER LipSat sensor processing in **(C)**. The percentage of p120* and p90* relative to total amount of sensor was quantified. The mean value and standard deviation from 3 biological replicates are depicted.

(E) Live imaging of *mga2*Δ cells expressing the INM LipSat sensor alone, or the ER LipSat alone or both LipSat sensors together. Genomically integrated Ole1-BFP was expressed from the strong *GPD* (*TDH3*) promoter. Peripheral endoplasmic reticulum, pER; nuclear envelope, NE; inner nuclear membrane, INM. Scale bar, 2 μm.

(F) Immunoblotting analysis of ER LipSat sensor processing in **(E)**. Pgk1 serves as a loading control.

(G) Immunoblotting analysis of INM LipSat sensor processing in **(E)**. Note that the mCh-tagged Heh2-p120*/p90* fragments have a higher molecular weight than p120/p90. Pgk1 serves as a loading control.

(H) Live imaging of cells expressing Ole1-mCherry on top of the wild-type allele. Ole1 was expressed from its endogenous or a strong *GPD* promoter. LDs are stained with BODIPY. Peripheral ER, pER; nuclear envelope, NE. Scale bar, 2 μm.

(I) Quantification of total LD number per cell in **(H)**. n = number of analyzed cells for each condition from 3 biological replicates. Mean value and standard deviation are indicated.

(J) Quantification of total LD volume per cell in **(H)** n = number of analyzed cells for each condition from 3 biological replicates. Mean value and standard deviation are indicated.

(K) Immunoblotting analysis of Ole1-mCherry expression levels in **(H)**. Pgk1 serves as a loading control.

(L) Immunoblotting analysis of Mga2-mCherry expression levels in **Figure 3A**. Pgk1 was used as a loading control.

(M) Automated quantification of total LD volume in **Figure 3A**. n = number of analyzed cells from 3 biological replicates. p-value (***<0.001) was determined by Mann-Whitney test. Mean value and standard deviation are indicated.

(N) Live imaging of wild-type cells expressing the indicated plasmid-based constructs from the strong *GPD* promoter. LDs are stained with BODIPY. Peripheral endoplasmic reticulum, pER; nuclear envelope, NE. Scale bar, 2 μm.

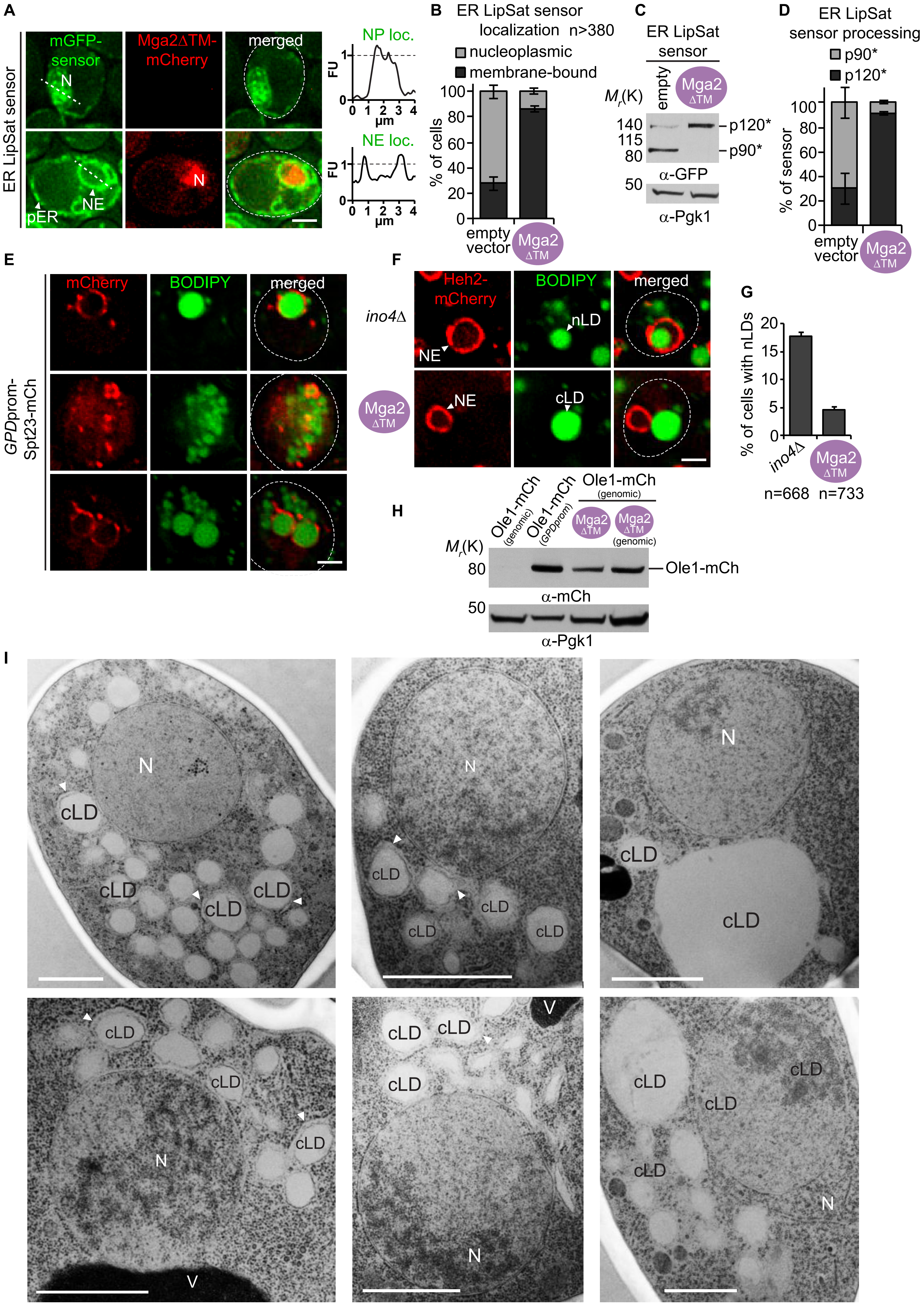
A
ER LipSat sensor
mGFP-sensor
Mga2ΔTM-mCherry
merged
N
NE
pER
NP loc.
NE loc.
FU
μm
B
ER LipSat sensor localization n>380
nucleoplasmic
membrane-bound
% of cells
empty vector
Mga2 ΔTM
C
ER LipSat sensor
empty
Mga2 ΔTM
Mr(K)
140
115
80
50
p120*
p90*
α-GFP
α-Pgk1
D
ER LipSat sensor processing
p90*
p120*
% of sensor
empty vector
Mga2 ΔTM
E
mCherry
BODIPY
merged
GPDprom-Spt23-mCh
F
ino4Δ
Mga2 ΔTM
Heh2-mCherry
BODIPY
merged
NE
nLD
cLD
G
% of cells with nLDs
ino4Δ
Mga2 ΔTM
n=668
n=733
H
Ole1-mCh (genomic)
Ole1-mCh (GPDprom)
Ole1-mCh (genomic)
Mga2 ΔTM
Mga2 ΔTM (genomic)
Mr(K)
80
50
Ole1-mCh
α-mCh
α-Pgk1
I
N
cLD
V

Figure S3

Supplementary Figure 3. Mga2 activity increases cytoplasmic LD formation, Related to Figure 3.

(A) Live imaging of the ER LipSat sensor co-expressed with Mga2ΔTM-mCherry or an empty vector. Genomically integrated Mga2ΔTM-mCherry was expressed from the strong *GPD* promoter in *mga2Δ* cells. Sensor fluorescence intensity was quantified across a line spanning the nucleus. For comparison the FU value 1 is marked with a horizontal dashed line. Arbitrary Fluorescence Units, FU; nucleus, N; peripheral endoplasmic reticulum, pER; nuclear envelope, NE; nucleoplasmic localization, NP loc; nuclear envelope localization, NE loc. Scale bar, 2 μm.

(B) Quantification of ER LipSat sensor localization in **(A)**. Phenotypes were classified as membrane-bound or nucleoplasmic. Mean value and standard deviation are depicted. n = number of analyzed cells for each condition from 3 biological replicates.

(C) Immunoblotting analysis of ER LipSat sensor processing in **(A)**. Pgk1 serves as a loading control.

(D) Quantification of ER LipSat sensor processing in **(C)**. The percentage of p120* and p90* relative to total amount of sensor was quantified. The mean value and standard deviation from 3 biological replicates are depicted.

(E) Overexpression of Spt23 induces LDs. Live imaging of wild-type cells expressing plasmid-based Spt23 from the strong *GPD* promoter. LDs are stained with BODIPY. Scale bar, 2 μm.

(F) Comparison of nLD content in *ino4Δ* or Mga2ΔTM overexpressing cells. Heh2-mCherry was used as an INM marker. LDs are stained with BODIPY. Cytoplasmic lipid droplet, cLD; nuclear lipid droplet, nLD; nuclear envelope, NE. Scale bar, 2 μm.

(G) nLD quantification in **(F)**. nLDs were defined as BODIPY-positive structures within the Heh2-labeled INM. More than 660 cells were counted for each condition from 3 biological replicates. The mean value and standard deviation are depicted.

(H) Expression levels of Ole1 under different experimental conditions. Ole1 was tagged genomically and upregulated via Mga2ΔTM overexpression or expressed from a strong *GPD* promoter. LDs in Mga2ΔTM-expressing cells are larger/more abundant (compare LD volume per cell in **Figures 3F** and **S2J**) even though Ole1 protein levels were not higher compared to Ole1 overexpressing cells. The likely reason for this effect is that Mga2 affects several target genes as shown in **Figure 4B** (e.g. *MVD1, PHS1*, *ICT1* and *ALE1*) which may synergize in buffering excess UFAs via LD biogenesis. Pgk1 serves as a loading control.

(I) TEM analysis of Mga2ΔTM overexpressing cells. A plasmid-based Mga2ΔTM construct was expressed from the *GPD* promoter in *mga2Δ* cells. ER membranes are

frequently wrapped around cLDs in this mutant (arrowheads). Nucleus, N; vacuole, V; cytoplasmic lipid droplet, cLD. Scale bar, 1 μm.

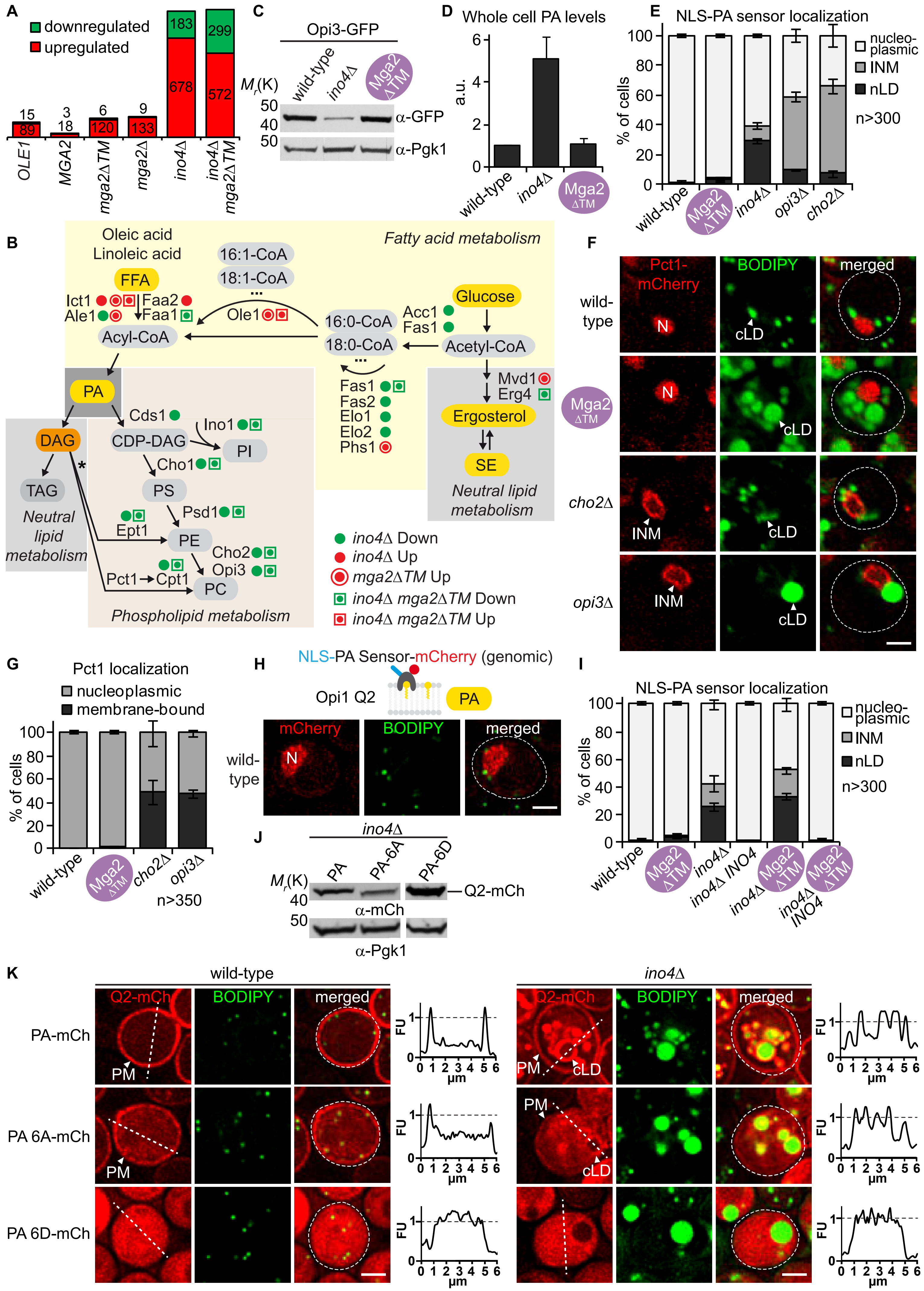
A
downregulated
upregulated
OLE1
MGA2
mga2ΔTM
mga2Δ
ino4Δ
ino4Δ mga2ΔTM
C
Opi3-GFP
wild-type
ino4Δ
Mga2 ΔTM
Mr(K)
50
40
50
α-GFP
α-Pgk1
D
Whole cell PA levels
a.u.
E
NLS-PA sensor localization
% of cells
nucleo-plasmic
INM
nLD
n>300
wild-type
opi3Δ
cho2Δ
B
Oleic acid
Linoleic acid
FFA
16:1-CoA
18:1-CoA
Fatty acid metabolism
Ict1
Ale1
Faa2
Faa1
Ole1
Acyl-CoA
16:0-CoA
18:0-CoA
Acc1
Fas1
Glucose
Acetyl-CoA
PA
Cds1
Ino1
CDP-DAG
PI
DAG
TAG
Cho1
PS
Psd1
Ept1
PE
Cho2
Opi3
Pct1
Cpt1
PC
Neutral lipid metabolism
Phospholipid metabolism
Fas1
Fas2
Elo1
Elo2
Phs1
Mvd1
Erg4
Ergosterol
SE
Neutral lipid metabolism
ino4Δ Down
ino4Δ Up
mga2ΔTM Up
ino4Δ mga2ΔTM Down
ino4Δ mga2ΔTM Up
F
Pct1-mCherry
BODIPY
merged
wild-type
Mga2 ΔTM
cho2Δ
opi3Δ
N
cLD
INM
G
Pct1 localization
nucleoplasmic
membrane-bound
% of cells
n>350
H
NLS-PA Sensor-mCherry (genomic)
Opi1 Q2
PA
mCherry
BODIPY
merged
wild-type
I
NLS-PA sensor localization
nucleo-plasmic
INM
nLD
n>300
ino4Δ INO4
J
ino4Δ
PA
PA-6A
PA-6D
Q2-mCh
α-mCh
α-Pgk1
K
wild-type
ino4Δ
Q2-mCh
BODIPY
merged
PA-mCh
PA 6A-mCh
PA 6D-mCh
PM
cLD
FU
μm

Figure S4

Supplementary Figure 4. Transcriptome analysis and cellular phosphatidylcholine / phosphatidic acid measurements, Related to Figure 4.

(A) Number of differentially transcribed genes in **Figure 4A**.

(B) Simplified scheme of lipid metabolism in yeast. Major pathways are color-coded and key lipid intermediates/end products are depicted. Differentially transcribed enzymes in the mutant strains are shown and marked with a green dot (down), red dot (up). Asterisk indicates the Kennedy pathway, which uses exogenous choline and ethanolamine together with DAG to form PE and PC.

(C) Immunoblotting analysis of Opi3-GFP protein levels confirms the transcriptional downregulation of *OPI3* in *ino4Δ* cells but not in Mga2ΔTM overexpressing cells (see **Figure 4B**). Pgk1 serves as a loading control.

(D) Whole cell analysis of phosphatidic acid (PA) levels in the indicated mutants compared to wild-type cells. Mean value and standard deviation for each condition from 3 experiments depicted. Arbitrary unit, a.u.

(E) Quantification of NLS-PA-mCherry sensor localization as observed in **Figure 4C**. n = number of analyzed cells from 3 biological replicates.

(F) Live imaging of Pct1-mCherry (genomically tagged) in the indicated strains. Cells were grown to stationary phase. Nucleus, N; inner nuclear membrane, INM; cytoplasmic lipid droplet, cLD. Scale bar, 2 μm.

(G) Quantification of Pct1 localization as observed in **(F)**. Phenotypes were classified as membrane-bound or nucleoplasmic. Mean value and standard deviation are depicted. n = number of analyzed cells for each condition from 3 biological replicates.

(H) Live imaging of wild-type cells expressing genomically integrated NLS-PA-mCherry sensor (control for **Figure 4E**). BODIPY stains LDs. Nucleus, N. Scale bar, 2 μm.

(I) Quantification of NLS-PA sensor localization in **Figure 4E**. n = number of analyzed cells from 3 biological replicates.

(J) Immunoblotting analysis of wild-type and mutant PA sensor expression levels (tagged with mCherry). Pgk1 serves as a loading control. See further explanations in **(K)**.

(K) PA sensing by the Opi1-derived PA sensor depends on specific residues within a critical amphipathic helix (AH). This AH harbors two PA-selective three-finger grips, each formed by three basic residues on one side of the helix. The first motif is a KRK motif and the second a 3K motif (Hofbauer et al., 2018). Opi1 harbors additional residues that contribute to PA recognition and these are also present in the sensor (Loewen et al., 2004; Romanauska and Kohler, 2018), but were not examined here.

We created mutations in the AH motifs to test whether sensor binding to PA-rich membranes in general, and binding to PA-rich LDs in particular would be affected. To this end the KRK and 3K motifs in our sensor (i.e. the Q2 domain of Opi1) were substituted by alanines (KRK & 3K > 6A; K112, R115, K119, K121, K125, K128). This variant forms a predicted AH and its mean helical hydrophobic moment (μH=0.289) is similar to wild-type (μH=0.357). This mutant sensor had a decreased affinity for PA-rich membranes in cells: labeling of PA-rich LDs as well as the PA-rich plasma membrane was reduced when tested in wild-type and *ino4*Δ cells. The residual binding to PA-rich membranes is explained by the contribution of residues outside of the AH. Mutating these on top of AH residues (Romanauska and Kohler, 2018) or a charge inversion of R/K residues to aspartate (6D mutant) (μH=0.344) further reduces membrane binding. Representative images of PA sensor mutants expressed in wild-type or *ino4*Δ cells. BODIPY stains LDs. For comparison the FU value 1 is marked with a horizontal dashed line. Arbitrary Fluorescence Units, FU; plasma membrane, PM; cytoplasmic lipid droplet, cLD. Scale bar, 2 μm.

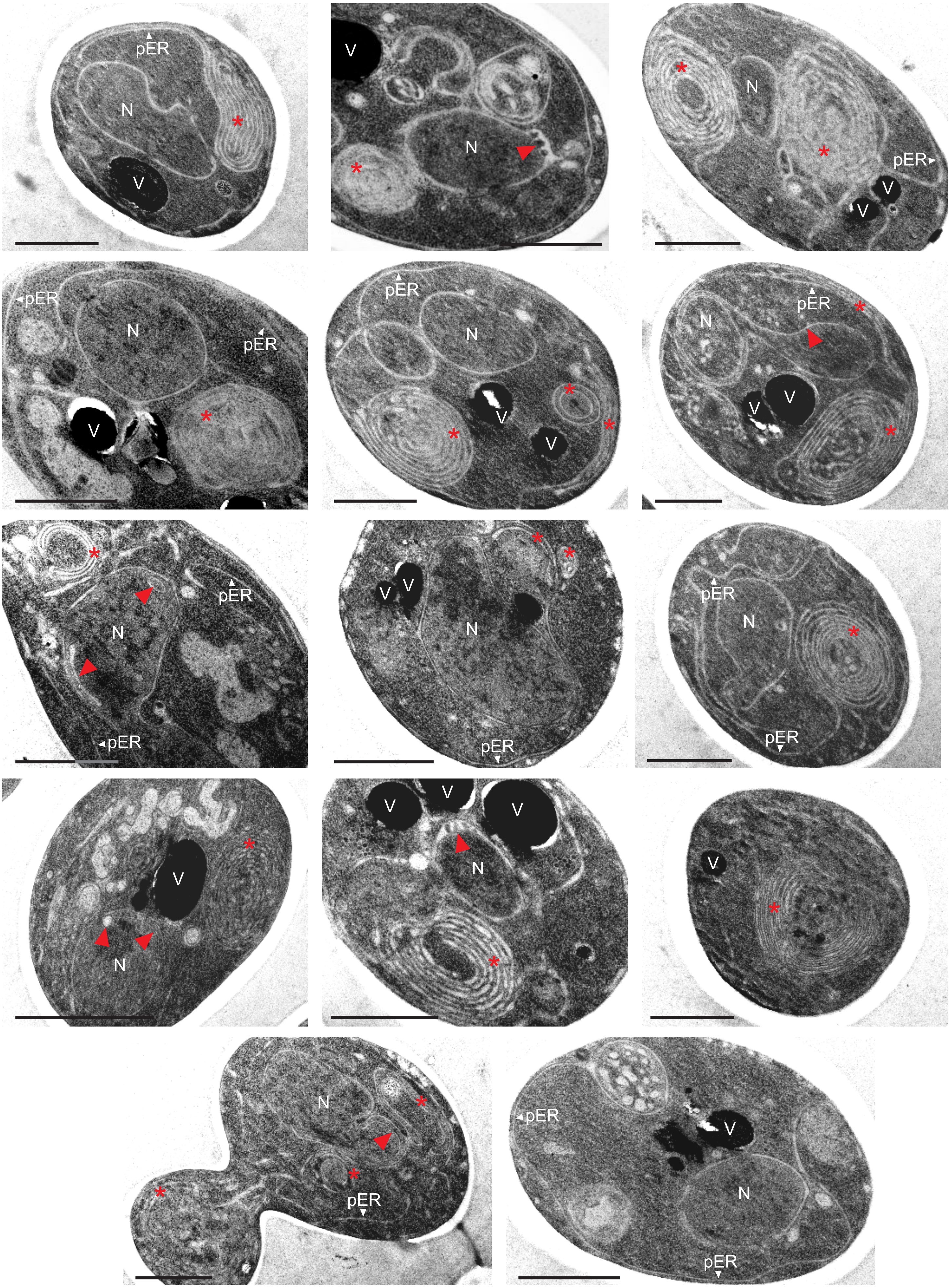

Figure S5

Supplementary Figure 5. High UFA levels cause ER and NE morphology defects in LD-deficient cells, Related to Figure 5.

TEM analysis of representative examples of *4*Δ cells overexpressing Mga2ΔTM from the inducible *GAL1* promoter. Nucleus, N; vacuole, V; peripheral endoplasmic reticulum, pER. Red asterisk marks membrane stacks/whorls; red arrowhead indicates NE defects including NE expansions and alterations of the perinuclear space. Scale bar, 1 μm.

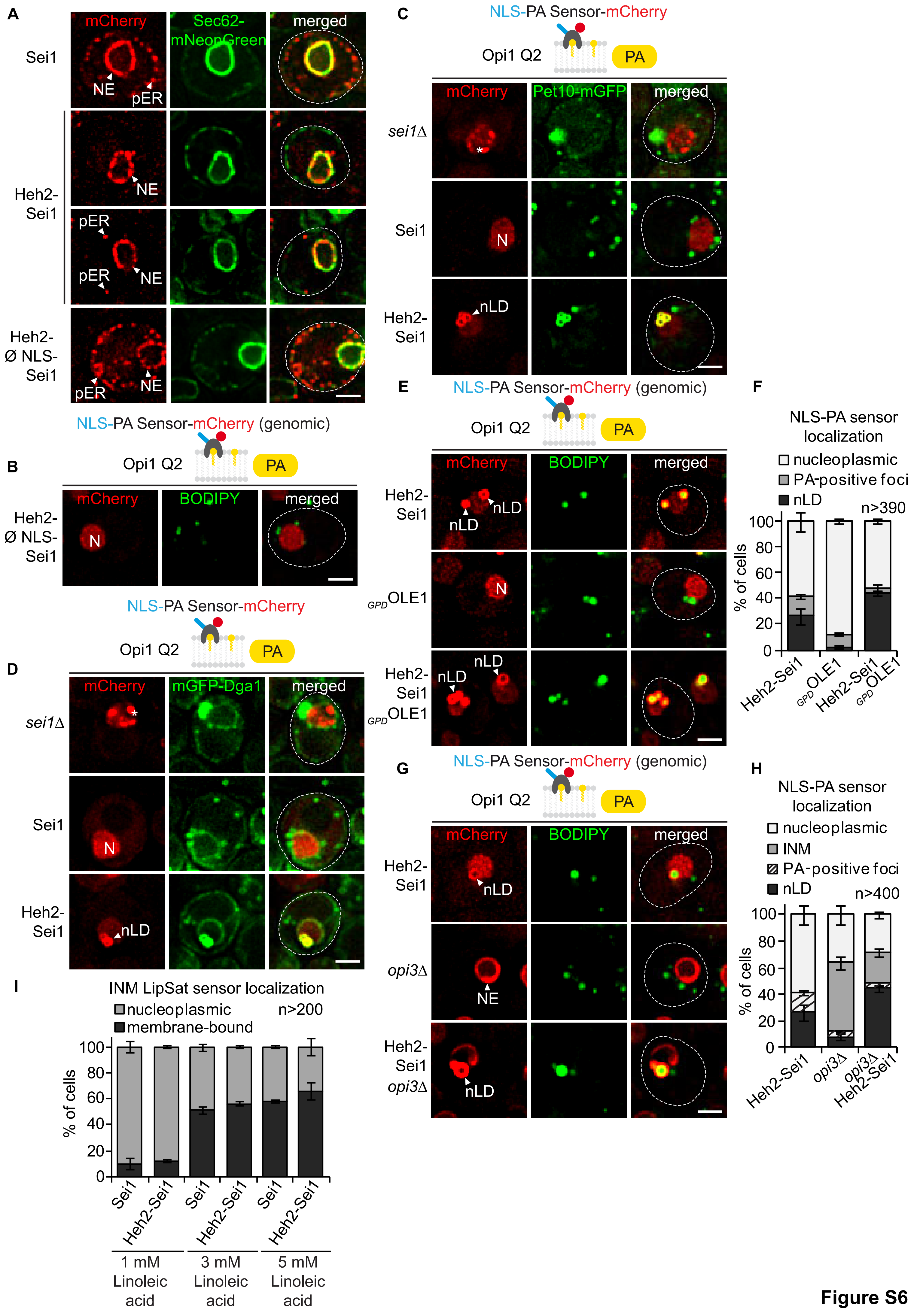
A
mCherry
Sec62-mNeonGreen
merged
Sei1
NE
pER
Heh2-Sei1
Heh2-Ø NLS-Sei1
B
NLS-PA Sensor-mCherry (genomic)
Opi1 Q2
PA
BODIPY
N
C
NLS-PA Sensor-mCherry
Pet10-mGFP
sei1Δ
nLD
D
mGFP-Dga1
E
$_{GPD}$OLE1
F
NLS-PA sensor localization
nucleoplasmic
PA-positive foci
nLD
n>390
% of cells
G
opi3Δ
Heh2-Sei1 opi3Δ
H
INM
n>400
I
INM LipSat sensor localization
membrane-bound
n>200
1 mM Linoleic acid
3 mM Linoleic acid
5 mM Linoleic acid

Figure S6

Supplementary Figure 6. Targeting Sei1 activity to the INM drives nLD production, Related to Figure 6.

(A) Live imaging of *sei1*Δ cells expressing the indicated *SEI1* constructs from the *SEI1* promoter and Sec62-mNeonGreen as an ER marker. The appended Heh2 fragment comprises an NLS (Heh2 aa93-137) and an adjacent linker (aa 138-317). Heh2-∅NLS-Sei1 indicates a Heh2 fragment with the omitted NLS. Nuclear envelope, NE; peripheral endoplasmic reticulum, pER. Scale bar, 2 μm.

(B) Live imaging of *sei1*Δ cells expressing the NLS-PA-mCherry sensor and the plasmid-based Heh2-∅NLS-Sei1 (*SEI1* promoter). Heh2-∅NLS-Sei1 indicates a Heh2 fragment with the omitted NLS. See also **Figure 6A**. BODIPY stains LDs. Nucleus, N. Scale bar, 2 μm.

(C) Live imaging of cells expressing the perilipin Pet10-mGFP*,* the NLS-PA-mCherry sensor, and the indicated *SEI1* constructs. *SEI1* constructs were expressed from the endogenous *SEI1* promoter in *sei1*Δ cells. Nucleus, N; nuclear lipid droplet, nLD. Asterisk marks PA-positive foci. Scale bar, 2 μm.

(D) Live imaging of cells expressing mGFP-Dga1, NLS-PA-mCherry sensor, and the indicated *SEI1* constructs. *SEI1* constructs were expressed from the endogenous *SEI1* promoter in *sei1*Δ cells. Nucleus, N; nuclear lipid droplet, nLD. Asterisk marks PA-positive foci. Scale bar, 2 μm.

(E) Live imaging of NLS-PA-mCherry sensor in the indicated strains as a readout for nLD production. nLDs have a BODIPY-positive core surrounded by a PA-rich shell. Nucleus, N; nuclear lipid droplet, nLD. Scale bar, 2 μm.

(F) Quantification of NLS-PA sensor localization in **(E)**. n = number of analyzed cells from 3 biological replicates.

(G) Live imaging of NLS-PA-mCherry sensor in the indicated strains as a readout for nLD production. nLDs have a BODIPY-positive core surrounded by a PA-rich shell. Nuclear envelope, NE; nuclear lipid droplet, nLD. Scale bar, 2 μm.

(H) Quantification of NLS-PA sensor localization in **(G)**. n = number of analyzed cells from 3 biological replicates.

(I) Quantification of INM LipSat sensor localization in **Figure 6L**. Phenotypes were classified as membrane-bound or nucleoplasmic. Mean value and standard deviation are depicted. n = number of analyzed cells for each condition from 3 biological replicates.

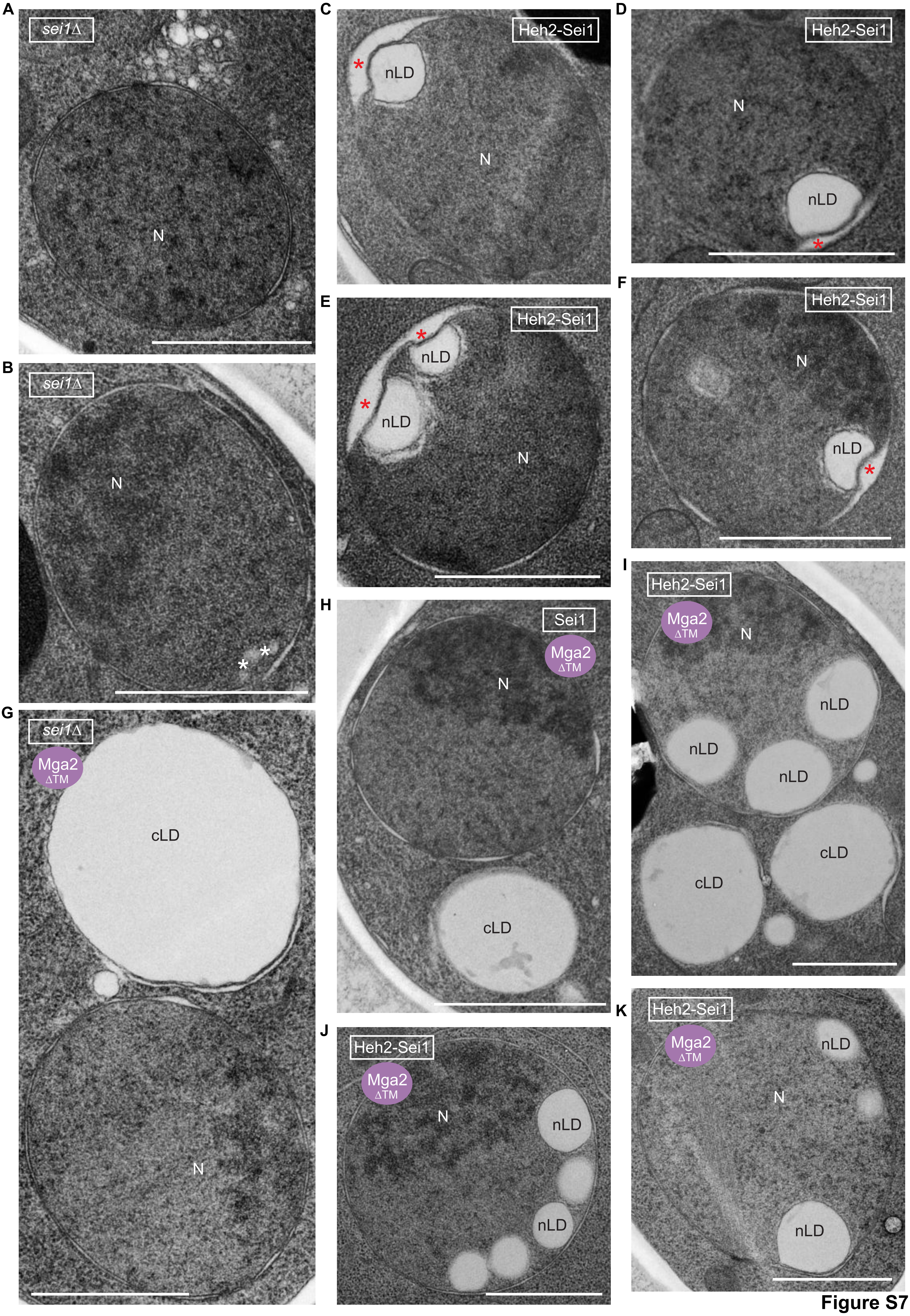

A
sei1Δ
N
B
sei1Δ
N
*
*
C
Heh2-Sei1
*
nLD
N
D
Heh2-Sei1
N
nLD
*
E
Heh2-Sei1
*
nLD
*
nLD
N
F
Heh2-Sei1
N
nLD
*
G
sei1Δ
Mga2 ΔTM
cLD
N
H
Sei1
Mga2 ΔTM
N
cLD
I
Heh2-Sei1
Mga2 ΔTM
N
nLD
nLD
nLD
cLD
cLD
J
Heh2-Sei1
Mga2 ΔTM
N
nLD
nLD
K
Heh2-Sei1
Mga2 ΔTM
nLD
N
nLD

Figure S7

Supplementary Figure 7. Ultrastructural analysis of Sei1-induced nLDs, Related to Figure 6.

(A-B) TEM analysis of representative examples of *sei1*Δ cells transformed with an empty vector. Nucleus, N. White asterisk indicates some droplet-like structures that might correspond to the small PA-positive foci observed by fluorescence microscopy in **Figure 6A**. Scale bar, 1 μm.

(C-F) TEM analysis of representative examples of Heh2-Sei1 expressing cells. Plasmid-based Heh2-Sei1 was expressed from the *SEI1* promoter in a *sei1*Δ strain. Nucleus, N; nuclear lipid droplet, nLD. Red asterisk marks a widened perinuclear space beneath an nLD. Scale bar, 1 μm.

(G) TEM analysis of a representative example of genomically integrated Mga2ΔTM in *sei1*Δ cells. Mga2ΔTM was expressed from the strong *GPD* promoter. Nucleus, N; cytoplasmic lipid droplet, cLD. Scale bar, 1 μm.

(H) TEM analysis of a representative example of Sei1 expression in genomically integrated Mga2ΔTM cells. Mga2ΔTM was expressed from the strong *GPD* promoter. Nucleus, N; cytoplasmic lipid droplet, cLD. Scale bar, 1 μm.

(I-K) TEM analysis of Heh2-Sei1 expression in genomically integrated Mga2ΔTM cells. Mga2ΔTM was expressed from the strong *GPD* promoter. Nucleus, N; cytoplasmic lipid droplet, cLD; nuclear lipid droplet, nLD. Scale bar, 1 μm.

Table S1. Yeast strains used in this study, related to the Key Resources Table and STAR Methods.

YEAST STRAINS	SOURCE	IDENTIFIER
S. cerevisiae strain *wild-type* (BY4741), genotype: *MATa; ura3Δ0; leu2Δ0; his3Δ1; met15Δ0*	Euroscarf	Y00000
S. cerevisiae strain *mga2Δ*, genotype: *MATa; ura3Δ0; leu2Δ0; his3Δ1; met15Δ0; mga2Δ::kanMX4*	Euroscarf	Y05968
S. cerevisiae strain *ino4Δ*, genotype: *MATa; ura3Δ0; leu2Δ0; his3Δ1; met15Δ0; ino4Δ::kanMX4*	Euroscarf	Y06258
S. cerevisiae strain *mga2Δ* Heh2-mCherry Mga2ΔTM, genotype: *MATa; ura3Δ0; leu2Δ0; his3Δ1; met15Δ0; mga2Δ::kanMX4; Heh2-mCherry::natNT2; GPDprom-mga2ΔTM-5xGS-VC::URA3*	This paper	N/A
S. cerevisiae strain *mga2Δ* Mga2ΔTM-mCherry, genotype: *MATa; ura3Δ0; leu2Δ0; his3Δ1; met15Δ0; mga2Δ::kanMX4; GPDprom-mga2ΔTM-mCherry::URA3*	This paper	N/A
S. cerevisiae strain *mga2Δ* Mga2ΔTM, genotype: *MATa; ura3Δ0; leu2Δ0; his3Δ1; met15Δ0; mga2Δ::kanMX4; GPDprom-mga2ΔTM-5xGS-VC::URA3*	This paper	N/A
S. cerevisiae strain *ino4Δ* Heh2-mCherry, genotype: *MATa; ura3Δ0; leu2Δ0; his3Δ1; met15Δ0; ino4Δ::kanMX4; Heh2-mCherry::natNT2*	This paper	N/A
S. cerevisiae strain NLS-PA-mCh, genotype: *MATa; ura3Δ0; leu2Δ0; his3Δ1; met15Δ0; ADH1prom-NLS-PA-mCh::HIS3*	This paper	N/A
S. cerevisiae strain Mga2ΔTM NLS-PA-mCh, genotype: *MATa; ura3Δ0; leu2Δ0; his3Δ1; met15Δ0; ADH1prom-NLS-PA-mCh::HIS3; GPDprom-mga2ΔTM-5xGS-VC::URA3*	This paper	N/A
S. cerevisiae strain *ino4Δ* NLS-PA-mCh, genotype: *MATa; ura3Δ0; leu2Δ0; his3Δ1; met15Δ0; ino4Δ::natNT2; ADH1prom-NLS-PA-mCh::HIS3*	This paper	N/A
S. cerevisiae strain *opi3Δ* NLS-PA-mCh, genotype: *MATa; ura3Δ0; leu2Δ0; his3Δ1; met15Δ0; opi3Δ::natNT2; ADH1prom-NLS-PA-mCh::HIS3*	This paper	N/A
S. cerevisiae strain *cho2Δ* NLS-PA-mCh, genotype: *MATa; ura3Δ0; leu2Δ0; his3Δ1; met15Δ0; cho2Δ::natNT2; ADH1prom-NLS-PA-mCh::HIS3*	This paper	N/A
S. cerevisiae strain *ino4Δ* NLS-PA-mCh Mga2ΔTM, genotype: *MATa; ura3Δ0; leu2Δ0; his3Δ1; met15Δ0; ADH1prom-NLS-PA-mCh::HIS3; GPDprom-mga2ΔTM-5xGS-VC::URA3; ino4Δ::natNT2*	This paper	N/A
S. cerevisiae strain Pct1-mCh, genotype: *MATa; ura3Δ0; leu2Δ0; his3Δ1; met15Δ0; PCT1-mCh::natNT2*	This paper	N/A
S. cerevisiae strain *mga2Δ* Pct1-mCherry Mga2ΔTM, genotype: *MATa; ura3Δ0; leu2Δ0; his3Δ1; met15Δ0; mga2Δ::kanMX4; Pct1-mCherry::natNT2; GPDprom-mga2ΔTM-5xGS-VC::URA3*	This paper	N/A
S. cerevisiae strain *opi3Δ* Pct1-mCherry, genotype: *MATa; ura3Δ0; leu2Δ0; his3Δ1; met15Δ0; opi3Δ::kanMX4; Pct1-mCherry::natNT2*	This paper	N/A
S. cerevisiae strain *cho2Δ* Pct1-mCherry, genotype: *MATa; ura3Δ0; leu2Δ0; his3Δ1; met15Δ0; cho2Δ::kanMX4; Pct1-mCherry::natNT2*	This paper	N/A

S. cerevisiae strain Ole1-BFP NLS-PA-mCh, genotype: *MATa; ura3Δ0; leu2Δ0; his3Δ1; met15Δ0; ADH1prom-NLS-PA-mCh::HIS3; GPDprom-OLE1-BFP::URA3*	This paper	N/A
S. cerevisiae strain *mga2Δ* Ole1-BFP, genotype: *MATa; ura3Δ0; leu2Δ0; his3Δ1; met15Δ0; mga2Δ::kanMX4; GPDprom-OLE1-BFP::URA3*	This paper	N/A
S. cerevisiae strain Opi3-GFP, genotype: *MATa; ura3Δ0; leu2Δ0; his3Δ1; met15Δ0; OPI3-GFP::natNT2*	This paper	N/A
S. cerevisiae strain *ino4Δ* Opi3-GFP, genotype: *MATa; ura3Δ0; leu2Δ0; his3Δ1; met15Δ0; ino4Δ::kanMX4; OPI3-GFP::natNT2*	This paper	N/A
S. cerevisiae strain *mga2Δ* Opi3-GFP Mga2ΔTM, genotype: *MATa; ura3Δ0; leu2Δ0; his3Δ1; met15Δ0; mga2Δ::kanMX4; OPI3-GFP::natNT2; GPDprom-mga2ΔTM-5xGS-VC::URA3*	This paper	N/A
S. cerevisiae strain 4Δ, genotype: BY4742 *MATα; ura3Δ0; leu2Δ0; his3Δ1; lys2Δ0; are1Δ::kanMX4; are2Δ::kanMX4; dga1Δ::kanMX4; lro1Δ::kanMX4*	(Petschnigg et al., 2009)	N/A
S. cerevisiae strain Ole1-mCh, genotype: *MATa; ura3Δ0; leu2Δ0; his3Δ1; met15Δ0; OLE1-mCh::natNT2*	This paper	N/A
S. cerevisiae strain *mga2Δ* Ole1-mCh, genotype: *MATa; ura3Δ0; leu2Δ0; his3Δ1; met15Δ0; mga2Δ::kanMX4; OLE1-mCh::natNT2*	This paper	N/A
S. cerevisiae strain *mga2Δ* Ole1-mCh Mga2ΔTM, genotype: *MATa; ura3Δ0; leu2Δ0; his3Δ1; met15Δ0; mga2Δ::kanMX4; OLE1-mCh::natNT2; GPDprom-mga2ΔTM-5xGS-VC::URA3*	This paper	N/A
S. cerevisiae strain *sei1Δ*, genotype: *MATa; ura3Δ0; leu2Δ0; his3Δ1; met15Δ0; sei1Δ::kanMX4*	Euroscarf	Y05313
S. cerevisiae strain *sei1Δ* NLS-PA-mCh, genotype: *MATa; ura3Δ0; leu2Δ0; his3Δ1; met15Δ0; sei1Δ::kanMX4; ADH1prom-NLS-PA-mCh::HIS3*	This paper	N/A
S. cerevisiae strain *sei1Δ opi3Δ* NLS-PA-mCh, genotype: *MATa; ura3Δ0; leu2Δ0; his3Δ1; met15Δ0; sei1Δ::kanMX6; opi3Δ::natNT2; ADH1prom-NLS-PA-mCh::HIS3*	This paper	N/A
S. cerevisiae strain *sei1Δ* NLS-PA-mCh Ole1-BFP, genotype: *MATa; ura3Δ0; leu2Δ0; his3Δ1; met15Δ0; sei1Δ::kanMX4; ADH1prom-NLS-PA-mCh::HIS3; GPDprom-OLE1-BFP::URA3*	This paper	N/A
S. cerevisiae strain *sei1Δ* NLS-PA-mCh Mga2ΔTM, genotype: *MATa; ura3Δ0; leu2Δ0; his3Δ1; met15Δ0; sei1Δ::kanMX4; ADH1prom-NLS-PA-mCh::HIS3; GPDprom-mga2ΔTM-5xGS-VC::URA3*	This paper	N/A
S. cerevisiae strain *sei1Δ mga2Δ*, genotype: *MATa; ura3Δ0; leu2Δ0; his3Δ1; met15Δ0; mga2Δ::kanMX4; sei1Δ::natNT2*	This paper	N/A
S. cerevisiae strain *sei1Δ mga2Δ* Ole1-BFP, genotype: *MATa; ura3Δ0; leu2Δ0; his3Δ1; met15Δ0; mga2Δ::kanMX4; sei1Δ::natNT2; GPDprom-OLE1-BFP::URA3*	This paper	N/A

Table S2. Plasmids used in this study, related to the Key Resources Table and STAR Methods.

PLASMIDS	SOURCE	IDENTIFIER
Yeast plasmids based on the pRS31X series	(Sikorski and Hieter, 1989)	N/A
Plasmid: *MGA2*prom-Mga2-mCh: *pRS313-MGA2prom-MGA2-mCh*	This paper	N/A
Plasmid: *MGA2*prom-Mga2ΔTM-mCh: *pRS313-MGA2prom-MGA2(1-1037)-mCh*	This paper	N/A
Plasmid: *GPD*prom-Mga2-mCh: *pRS316-GPDprom-MGA2-mCh*	This paper	N/A
Plasmid: *GPD*prom-Mga2ΔTM-mCh: *pRS316-GPDprom-MGA2(1-1037)-mCh*	This paper	N/A
Plasmid: *GAL1*prom-Mga2ΔTM-mCh: *pRS316-GAL1prom-MGA2(1-1037)-mCh*	This paper	N/A
Plasmid: *GPD*prom-Mga2ΔTM-5xGS-VC: *pRS306-GPDprom-MGA2(1-1037)-5xGS-VC*	This paper	N/A
Plasmid: *GPD*prom-Mga2ΔTM-5xGS-VC: *pRS316-GPDprom-MGA2(1-1037)-5xGS-VC*	This paper	N/A
Plasmid: *GPD*prom-Mga2ΔTM-mCh: *pRS306-GPDprom-MGA2(1-1037)-mCherry*	This paper	N/A
Plasmid: *ER LipSat sensor*: *pRS313-ADH1prom-mGFP-MGA2(128-1113)*	This paper	N/A
Plasmid: *ER LipSat sensor*: *pRS315-ADH1prom-mGFP-MGA2(128-1113)*	This paper	N/A
Plasmid: *ER LipSat sensor-mCh*: *pRS313-ADH1prom-mGFP-MGA2(128-1113)-mCherry*	This paper	N/A
Plasmid: *INM LipSat sensor*: *pRS313-ADH1prom-mGFP-HEH2(93-317)-MGA2(128-1113)*	This paper	N/A
Plasmid: *INM W1042A LipSat sensor mutant*: *pRS313-ADH1prom-mGFP-HEH2(93-317)-MGA2(128-1113)W1042A*	This paper	N/A
Plasmid: *INM P1044L LipSat sensor mutant*: *pRS313-ADH1prom-mGFP-HEH2(93-317)-MGA2(128-1113)P1044L*	This paper	N/A
Plasmid: *INM LipSat sensor-mCh*: *pRS313-ADH1prom-mGFP-HEH2(93-317)-MGA2(128-1113)-mCherry*	This paper	N/A
Plasmid: mCh-*INM LipSat sensor*: *pRS313-ADH1prom-mCherry-HEH2(93-317)-MGA2(128-1113)*	This paper	N/A
Plasmid: mGFP-Mga2: *pRS313-ADH1prom-mGFP-MGA2*	This paper	N/A
Plasmid: *MGA2*prom-mGFP-Mga2: *pRS313-MGA2prom-mGFP-MGA2*	This paper	N/A
Plasmid: *GPD*prom-Ole1-mCh: *pRS316-GPDprom-OLE1-mCh*	This paper	N/A
Plasmid: *GAL1*prom-Ole1-mCh: *pRS316-GAL1prom-OLE1-mCh*	This paper	N/A
Plasmid: *OLE1*prom-Ole1-mCh: *pRS316-OLE1prom-OLE1-mCh*	This paper	N/A
Plasmid: *GPD*prom-Ole1-BFP: *pRS316-GPDprom-OLE1-BFP*	This paper	N/A
Plasmid: *GPD*prom-Ole1-BFP: *pRS306-GPDprom-OLE1-BFP*	This paper	N/A
Plasmid: NLS-PA-mCh: *pRS303-ADH1prom-NUP60(1-24)-OPI1(103-191)-mCh*	This paper	N/A
Plasmid: NLS-PA-mCh: *pRS316-CYC1prom-NUP60(1-24)-OPI1(103-191)-mCh*	(Romanauska and Kohler, 2018)	N/A

Plasmid: *GPD*prom-Spt23-mCh: *pRS316-GPDprom-SPT23-mCh*	This paper	N/A
Plasmid: PA-mCh: *pRS316-CYC1prom-OPI1(103-191)-mCh*	(Romanauska and Kohler, 2018)	N/A
Plasmid: *INO4prom*-INO4: *pRS315-INO4prom-INO4*	This paper	N/A
Plasmid: *GPD*prom-Faa4-mCh: *pRS316-GPDprom-FAA4-mCh*	This paper	N/A
Plasmid: *GPD*prom-Elo1-mCh: *pRS316-GPDprom-ELO1-mCh*	This paper	N/A
Plasmid: *GPD*prom-Mvd1-mCh: *pRS316-GPDprom-MVD1-mCh*	This paper	N/A
Plasmid: Sei1: *pRS315-SEI1prom-mGFP-SEI1*	This paper	N/A
Plasmid: Heh2-Sei1: *pRS315-SEI1prom-mGFP-HEH2(93-317)-SEI1*	This paper	N/A
Plasmid: Heh2-Sei1: *pRS313-SEI1prom-mGFP-HEH2(93-317)-SEI1*	This paper	N/A
Plasmid: Heh2∅NLS-Sei1: *pRS315-SEI1prom-mGFP-HEH2(138-317)-SEI1*	This paper	N/A
Plasmid: Sec62-mNeonGreen: *pRS316-SEC62prom-SEC62-5xGS-mNeonGreen*	This paper	N/A
Plasmid: Pet10-mGFP: *pRS313-PET10prom-PET10-mGFP*	This paper	N/A
Plasmid: mGFP-Dga1: *pRS313-ADH1prom-mGFP-DGA1*	This paper	N/A
Plasmid: PA 6A-mCh: *pRS316-CYC1prom-opi1(103-191)K112A R115A K119A K121A K125A K128A-mCh*	This paper	N/A
Plasmid: PA 6D-mCh: *pRS316-CYC1prom-opi1(103-191)K112D R115D K119D K121D K125D K128D-mCh*	This paper	N/A
